# Supplementary material for: HABIT: Health visitors delivering Advice in Britain on Infant Toothbrushing – an early-phase feasibility study of a complex oral health intervention
Source: BMJ Open. 2022 Oct 10;12(10):e059665. doi: 10.1136/bmjopen-2021-059665 (PMC9557312; doi:10.1136/bmjopen-2021-059665)
Supplement: Supplementary data [file bmjopen-2021-059665supp002.pdf]

Supplementary Item 2 – Dietary Data

Frequency of food and drinks averaged for all participants across the 3 data collection visits, coded using the following key: 0 - None; 1 - less than once a month; 2 - 1-3 time per month; 3 – once per week; 4 – 2-4 times per week; 5 – 5-6 times per week; 6 once per day; 7 – 2-3 times per day; 8 – 4-5 times per day; 9 – 6 or more times a day

	Median Frequency		
	Baseline (n=27)	2 Week Follow-Up (n=26)	3 Month Follow-Up (n=26)
Water	7	8	7
Milk	7	7	7
Sugared drinks	0	0	0
Cakes	0	0	0
Biscuits	4	4	2
Sweets	0	0	1
Fresh Fruit	7	7	7
Vegetables	7	7	6
