# Supplementary material for: HABIT: Health visitors delivering Advice in Britain on Infant Toothbrushing – an early-phase feasibility study of a complex oral health intervention
Source: BMJ Open. 2022 Oct 10;12(10):e059665. doi: 10.1136/bmjopen-2021-059665 (PMC9557312; doi:10.1136/bmjopen-2021-059665)
Supplement: Author's manuscript [file bmjopen-2021-059665.draft_revisions.pdf]

BMJ Open is committed to open peer review. As part of this commitment we make the peer review history of every article we publish publicly available.

When an article is published we post the peer reviewers' comments and the authors' responses online. We also post the versions of the paper that were used during peer review. These are the versions that the peer review comments apply to.

The versions of the paper that follow are the versions that were submitted during the peer review process. They are not the versions of record or the final published versions. They should not be cited or distributed as the published version of this manuscript.

BMJ Open is an open access journal and the full, final, typeset and author-corrected version of record of the manuscript is available on our site with no access controls, subscription charges or pay-per-view fees (http://bmjopen.bmj.com).

If you have any questions on BMJ Open's open peer review process please email info.bmjopen@bmj.com

BMJ Open

HABIT: Health visitors delivering Advice in Britain on Infant Toothbrushing – An early-phase feasibility study of a complex oral health intervention

Journal:	*BMJ Open*
Manuscript ID	bmjopen-2021-059665
Article Type:	Original research
Date Submitted by the Author:	03-Dec-2021
Complete List of Authors:	Giles, Erin; University of Leeds, School of Dentistry<br>Wray, Faye; University of Leeds, Leeds Institute of Health Sciences<br>Eskyte, Ieva; University of Leeds, School of Law<br>Gray-Burrows, Kara ; University of Leeds, School of Dentistry<br>Owen, Jenny; University of Leeds, School of Dentistry<br>Bhatti, Amrit; University of Leeds, School of Dentistry<br>Zoltie, Tim; University of Leeds, School of Dentistry<br>McEachan, Rosemary; Bradford Institute for Health Research<br>Marshman, Z; University of Sheffield Faculty of Medicine Dentistry and Health<br>Pavitt, Sue; University of Leeds, School of Dentistry<br>West, Robert; University of Leeds, Leeds Institute of Health Sciences<br>Day, Peter; University of Leeds, School of Dentistry
Keywords:	PUBLIC HEALTH, Community child health < PAEDIATRICS, PREVENTIVE MEDICINE

SCHOLARONE™
Manuscripts

I, the Submitting Author has the right to grant and does grant on behalf of all authors of the Work (as defined in the below author licence), an exclusive licence and/or a non-exclusive licence for contributions from authors who are: i) UK Crown employees; ii) where BMJ has agreed a CC-BY licence shall apply, and/or iii) in accordance with the terms applicable for US Federal Government officers or employees acting as part of their official duties; on a worldwide, perpetual, irrevocable, royalty-free basis to BMJ Publishing Group Ltd ("BMJ") its licensees and where the relevant Journal is co-owned by BMJ to the co-owners of the Journal, to publish the Work in this journal and any other BMJ products and to exploit all rights, as set out in our licence.

The Submitting Author accepts and understands that any supply made under these terms is made by BMJ to the Submitting Author unless you are acting as an employee on behalf of your employer or a postgraduate student of an affiliated institution which is paying any applicable article publishing charge ("APC") for Open Access articles. Where the Submitting Author wishes to make the Work available on an Open Access basis (and intends to pay the relevant APC), the terms of reuse of such Open Access shall be governed by a Creative Commons licence – details of these licences and which Creative Commons licence will apply to this Work are set out in our licence referred to above.

Other than as permitted in any relevant BMJ Author's Self Archiving Policies, I confirm this Work has not been accepted for publication elsewhere, is not being considered for publication elsewhere and does not duplicate material already published. I confirm all authors consent to publication of this Work and authorise the granting of this licence.

For peer review only

HABIT: Health visitors delivering Advice in Britain on Infant Toothbrushing – An early-phase feasibility study of a complex oral health intervention

Authors

Erin Giles, Faye Wray, Ieva Eskyte, Kara A Gray-Burrows, Jenny Owen, Amrit Bhatti, Tim Zoltie, Rosemary RC McEachan, Zoe Marshman, Sue Pavitt, Robert West, Peter Day

Corresponding Author

Erin Giles – Department of Paediatric Dentistry, School of Dentistry, University of Leeds, Leeds, LS2 9LU. Email: e.giles@leeds.ac.uk

Keywords

Dental caries, prevention, behaviour change conversations, oral health habits, dental team, health visitors, intervention, parents, feasibility, quantitative

Trial Registration

ISRCTN55332414

Word Count

3997

For peer review only

Abstract (Word Count: 290)

Objectives: to conduct an early-phase feasibility study of an oral health intervention, HABIT, delivered by Health Visitors to parents of children aged 9-12-months-old.

Design: a mixed-methods, early-phase, non-controlled, feasibility study.

Participants: recruitment consisted of Group A – HABIT-trained Health Visitors (n=11), and Group B - parents of children aged 9-12-months-old about to receive their universal health check (n=35).

Setting: Bradford, West Yorkshire, United Kingdom

Intervention: Following a complex intervention methodology, our multi-disciplinary team co-developed digital and paper-based training and resources with health visitors and parents of young children. The intervention comprised of two components: (A) training for health visitors to deliver the HABIT intervention and (B) HABIT resources for parents, including a website, videos, toothbrushing demonstration and a paper-based leaflet with an action plan.

Primary and Secondary Outcome Measures: Recruitment, retention, and intervention delivery were analysed as key process outcomes. Group B demographics, self-reported toothbrushing behaviours, dietary habits and three objective measures of toothbrushing including plaque scores were collected at baseline, 2-weeks and 3-months post intervention.

Results: HABIT intervention delivery was feasible. Although the intended sample size was recruited, it was more challenging than anticipated. Retention of Group B participants to final data collection was satisfactory (n=26). Total compliance with toothbrushing guidelines at baseline was low (30%), but significantly improved and was maintained 3-months after the intervention (68%). Plaque scores improved post intervention and participants found video-recording of toothbrushing acceptable. Dietary habits remained largely unchanged.

Conclusion: This feasibility study has demonstrated that HABIT is an appropriate oral health intervention. Adaptions to the study design are recommended to maximise recruitment and data collection in a definitive study. These quantitative findings have demonstrated an early signal of impact for improved oral health behaviours for young children at high risk of decay.

Registration: ISRCTN55332414

For peer review only

Article Summary

Strengths and limitations of this study

- HABIT - a complex, co-designed oral health behavioural intervention – has been developed using MRC guidance.
- This early-phase feasibility study analysed recruitment, retention and intervention delivery as key process outcomes.
- Data were collected in the home setting and included clinical, observed and self-reported toothbrushing and dietary habit data collected at three time points: baseline and at two weeks and three months post HABIT intervention.
- Self-reporting of toothbrushing and dietary has limitations which the authors recognise.
- Challenges relating to recruitment and retention of participants will need to be addressed prior to progression to definitive study or full trial.

For peer review only

Background

Untreated dental caries is the most common health condition in the world. It is currently estimated that over half a billion children worldwide suffer from caries in their primary teeth [1]. In England, a quarter of children have experienced caries by the age of five [2]. However, wide disparities exist. Caries rates in deprived areas are around double the national average [2]. The sequalae of untreated dental disease is significant with clear consequences to the child, their family and wider society – negative consequences include pain, infection, time off school, time off work for parents and a poorer quality of life [3-5].

Caries is a public health priority owing to its prevalence and impact. Critically, however, it is preventable. Optimal home-based oral health habits established in early childhood provide long-term protective benefits [6]. National guidelines in England [7], provide clear, evidence-based definitions for these 'optimal' home-based oral health practices, including; brushing a child's teeth twice a day with a smear of at least 1000ppm fluoridated toothpaste as soon as the first tooth erupts into the mouth, and limiting the frequency and amount of sugary foods and drinks [7].

Health visiting teams in England provide a crucial role in giving children the best start possible in early-childhood by signposting families to support, encouraging positive health behaviours and safeguarding children at risk [8]. Five mandatory universal visits are undertaken by the health visiting team during the first two years of each child's life, the fourth visit takes place when the child is aged 9-12-months-old [9]. During this visit topics such as child development, health, nutrition, and obesity prevention are discussed. These visits can be undertaken by health visitors or appropriately trained nursery nurses. In this paper the term 'Health Visitors' will be used collectively to represent any health visiting team member who took part in the HABIT intervention.

The first 1000 days of a child's life are generally accepted to be the most important in their physical, psychological and emotional development [10 11]. As part of the Healthy Child Programme [12], oral health promotion is a key topic to be covered during these mandatory visits. The eruption of an infant's first tooth occurs at around six months of age [13], therefore the fourth universal health visit is usually the first opportunity for toothbrushing guidance to be discussed. Health Visitors, however, have identified barriers to provision of oral health advice including a lack of specialist knowledge, training, resources, management of resistant parents and recognising oral health as a priority [14-18].

"Health visitors delivering Advice in Britain on Infant Toothbrushing" (HABIT) is a co-designed complex oral health intervention, underpinned by behaviour change theory [19 20]. Feasibility studies are a critical step in the intervention development process; exploring acceptability, feasibility of delivery, recruitment and retention of participants and anticipating problems prior to a large-scale Randomised Control Trial (RCT) [21].

Aims and Objectives

For peer review only

The primary aim was to:

- Conduct an early-phase feasibility study of an oral health intervention, HABIT, delivered by Health Visitors to parents of children aged 9-12-months-old.

The objectives were to:

- Analyse recruitment and retention of participants of both Health Visitors and parents
- Assess the feasibility of intervention delivery and data collection
- Explore the potential impact of HABIT on optimal oral health behaviours

Method

Study design

This was a mixed-methods, early-phase, non-controlled, feasibility study. Health Visitors and Parents of children aged 9-12 months were recruited, all receiving the intervention.

Patient and Public Involvement

Participants were involved in the design, conduct, reporting and dissemination of this research project. Using a series of focus groups, previously designed health-visiting oral health resources were discussed and evaluated by Health Visitors and parents of young children. These discussions, in combination with the Medical Research Council (MRC) framework[22], informed the development of the intervention. A designated member of the research team supported and kept in regular contact with participants, allowing real-time feedback and alterations to the study conduct. Acceptability of the intervention and study design to participants is reported in a separate paper exploring the qualitative findings [23]. In addition, key stakeholders were invited to a research dissemination event held in Bradford in which preliminary findings were presented and discussed. Participation included representatives from: Health Visiting and dental teams, Bradford District Care NHS Foundation Trust, Bradford Metropolitan District Council and the British Society of Paediatric Dentistry, Oral Health Strategy Group and Public Health England.

Intervention outline

HABIT is an oral health intervention, underpinned by complex behavioural change theory, undertaken in a home setting. It comprises of two parts: 1) Training Health Visitors to deliver the intervention; 2) Delivery of the HABIT intervention by trained Health Visitors at the 9–12-month universal health visit. Oral health conversations are supported by HABIT resources for parents, including a website, videos, toothbrushing demonstration and a paper-based leaflet with an action

For peer review only

plan. A detailed summary of the HABIT intervention is outlined using the TIDieR checklist [24] in Supplementary Item 1.

Setting

Bradford is a metropolitan borough located in the United Kingdom (UK), situated within the northern county of West Yorkshire. It is the fifth largest city in the UK with a population of approximately half a million. Bradford's urban areas are among the most deprived in the UK [25]. Caries predominantly affects lower-socioeconomic areas of England therefore it is an ideal location for the implementation oral health research. The population of Bradford is also ethnically diverse – 64% of the population identify as White British, with 20% of the population of Pakistani ethnic-origin [26].

Recruitment & Retention

Group A participants were Health Visitors who provide the 9-12-month universal health check. They were invited to take part via email and those that volunteered to participate attended a one-day training course provided by the researchers.

Group B participants were recruited from the Bradford waiting list of children due to have their 9-12-month universal check. For each HABIT-trained Health Visitor, a report of their next 20 visits was identified. An invitation letter, project information sheet and consent form were posted with the standard universal check reminder letter. For Group B:

Inclusion criteria:

- Parents of a child aged 9-12-months-old who were about to receive a universal home visit by a HABIT-trained Health Visitor.
- Child with at least one erupted tooth

Exclusion criteria:

- Non-English-speaking families, as interpreter services were not available to the research team.

A research officer contacted parents by phone to explore if they wanted to participate. If the invitation was accepted, they would visit the parent to gain written consent and organise a home visit for baseline data collection. As suggested in good practice recommendations [27], we intended to recruit 30 parents-children (dyads) to our feasibility study. Thirty participants would provide a 95%

For peer review only

confidence interval (CI) for prevalence of no wider than ±19.6%, anticipating a minimum of 15% loss to follow-up.

Data collection

Data collection was carried out by three dental professionals experienced in providing care for children (SH, JO, HG), supported by three research assistants (KT, IE, FW). An experienced British Association of the Study of Community Dentistry (BASCD) examiner provided training to ensure a consistent approach to inspection procedures, tooth codes and diagnostic criteria [28]. Dental researchers underwent calibration for caries and plaque detection, with agreement calculated using Fleiss' kappa.

Data were collected in a home setting. Children were examined supine, using a disposable dental mirror and a head torch for illumination. Cotton rolls were available, if required, to remove debris. Between the baseline and second data collection visit, parents received the HABIT intervention delivered by a Health Visitor as part of the child's universal 9-12-month health visit. Further data collection was carried out two-weeks and three-months following the intervention. A flowchart of study design is outlined in Figure 1.

1) First visit – baseline (BL). Self-reported toothbrushing and dietary behaviours were collected through a questionnaire. Toothbrushing diaries were issued as an additional, contemporaneous, measure of toothbrushing frequency. Three objective measures of parental supervised brushing (PSB) were collected:

   a) Teeth were examined for cavitated dentinal caries and restorations using the BASCD criteria [28].
   b) Children's pre-brushing plaque levels per sextant using the Oral Hygiene Index [29]
   c) Duration of toothbrushing: researchers filmed parent-child toothbrushing for subsequent evaluation [30]

2) Second visit – two-week follow-up (2WFU)

   Two weeks following the HABIT intervention, further self-report questionnaire data and objective measures of PSB (a-c above) were collected at a home visit.

3) Third visit – three-month follow-up (3MFU)

   Three months following the HABIT intervention, further self-report questionnaire data and objective measures of PSB (a-c above) were collected at a home visit.

A £10 gift voucher was issued after each home-based data collection visit.

For peer review only

Self-reported oral health behaviours

The validated questionnaire[31 32] (see Appendix 1), collected information on participant sociodemographic data, self-reported toothbrushing, dietary behaviours and feeding practices. Self-report determinants of toothbrushing were measured against national guidance[7] outlining five key items for toothbrushing: parental supervision, 1000ppm toothpaste fluoride concentration, pea-sized amount of toothpaste, twice-daily toothbrushing and wiping away excess toothpaste at the end of brushing. A compound measure of 'total' compliance was also calculated. Contemporaneous data on the frequency of toothbrushing was collected by a paper diary. Dietary data were collected using an established questionnaire trialled within a Bradford birth cohort of similar aged children [33], measuring frequency of consumption of the main food groups. Frequency scoring was non-linear (e.g. 0=none, 1=less than once a month, etc.) and covered all aspects of diet with a focus on sugary snacks, sweetened beverages and fruit and vegetable intake.

Dental examination objective measures of toothbrushing

Teeth were examined for cavitated caries and dental restorations using the BASCD criteria [28]. A score was calculated based on the number of decayed, missing and filled teeth (dmft). Prior to performing the Oral Hygiene Index, allergy status was confirmed (checking no allergy to food colourings) and petroleum jelly was applied to the lips. The infants' teeth were dyed using TePe PlaqSearch™ disclosing solution applied with a small dental brush. Once the plaque index (score) had been taken, gauze was used to wipe the dyed plaque from the teeth to ensure it did not act as a visual aid for the parent in the subsequent videotaping of toothbrushing. Partially completed plaque scores were discounted in the statistical analysis.

Videotaping of child

Videotaping of child-parent toothbrushing was undertaken using a small action camera (GoPro HERO 5, Go Pro Inc). Practicability of video recordings pertains to the feasibility of data collection and was therefore analysed alongside toothbrushing duration. The videos also provided an objective method of assessment with national toothbrushing guidelines [7] and will be reported in a separate paper.

<u>Data Analysis</u>

Data relating to recruitment, retention, intervention delivery and feasibility of data collection were analysed using descriptive statistics in MS Excel. Progression criteria to definitive study were agreed: >25% recruitment and >75% retention - automatic progression; 20-25% recruitment and 65-74% retention - modifications recommended; <20% recruitment and <64% retention - consultation with advisory group and remedial actions required.

For peer review only

To explore potential oral health impacts, confidence intervals (CIs) for continuous variables (e.g. plaque scores) were calculated using a t-distribution. For categorical outcomes, the CIs for rate were calculated based on the binomial distribution. In respect to self-reported toothbrushing compliance, repeated measurements were fitted to a multi-level model with timepoints nested within dyads and allocating a random intercept for dyad. Maximum likelihood fitting was used rather than reduced maximum likelihood to permit hypothesis testing. Within the model, a term was added for 'time points' increasing the degrees of freedom by two. This enabled the reporting of compliance rates at each time point and formal statistical testing of the effect of the intervention over time.

The statistical significance of the time term was then determined by use of the log-likelihood ratio test. Similarly, a two-level linear regression was fitted for plaque scores and changes over time tested through a log-likelihood test. This analysis was valid under the assumption that drop out was at random. The analysis was undertaken in the R statistical software environment [34] and using the lme4 package [35].

Results

Recruitment and Retention

Eleven Health Visitors were recruited to Group A (n=11) to deliver the HABIT intervention. Of the eleven, eight (73%) including six health visitors and two nursery nurses, had the opportunity to deliver the intervention, as outlined in Figure 2.

Group B dyads were recruited over a 10-month period between December 2017 and October 2018. From 127 invitations, 35 dyads were recruited and consented - a recruitment rate of 28%. Anticipating a minimum of 15% loss to follow-up, the achieved sample size of n=35 was sufficient. Nine participants were lost, resulting in a retention rate of 74% (n=26). Almost all dropouts occurred between consent (n=8) and prior to baseline data collection. Figure 2 shows the participant flow and reasons for dropout for Group B participants.

Demographics

The baseline demographic data is shown in Table 1. There was significant variation in education, employment status and family group types. Almost half (n=12) of the dyads lived in the most deprived decile area of England, according to the Index of Multiple Deprivation (IMD) 2019 [25]. The average age of a parent in the study was 31, and the majority (n=21) were born in the United Kingdom. No child in the study had caries, an unsurprising finding given the age of the children.

For peer review only

Table 1: Baseline demographic data

Characteristic	(N=27)
Age of Parent	
Mean	31
Range	19, 42
% over 30	70%
Birthplace	
United Kingdom	78%
Pakistan	11%
Other	11%
Number of children	
Multi-child house	63%
Qualifications	
Less than 5 GCSEs	30%
A-level or equivalent	37%
University degree	33%
Employment	
Currently employed	48%
Household Income	
Less than £16100	11%
£16100 to £ 21249	26%
£21250 - £27999	4%
£28000 - £38399	7%
£38399 +	19%
Unanswered/Unsure	33%
Finances	
'Living Comfortably'	41%
'Doing Alright'	29%
'Just Getting By'	4%
'Finding It Difficult'	0%
Unanswered/Unsure	26%
IMD Centile	
Most Deprived	
1st	44%
2nd	7%
3rd	15%
4th	15%
5th	7%
6th	7%
7th	4%
8th	-
9th	-
10th	-
Least Deprived	

For peer review only

Feasibility of data collection and intervention delivery

Out of a possible 79 home visits, the research team were able to undertake 97% (n=77) of planned data collection. Reasons for the two missed visits are outlined in Figure 2. Of the 27 dyads who completed baseline data collection, all received the HABIT intervention.

Intervention Outcomes

Self-reported toothbrushing behaviours

For self-reported toothbrushing habits, 'total' compliance improved from 30% (n=27; 95%CI 0.13 to 0.47) at baseline to 70% (n=24; 95%CI 0.53 to 0.89) two-weeks and 68% (n=25; 95%CI 0.50 to 0.86) three-months following the HABIT intervention. These findings were statistically significant and are shown in Table 2. After the intervention, all parents had commenced toothbrushing. Compliance to all individual components of the guideline increased, albeit insignificantly, after the intervention and are outlined in Table 2. There was poor uptake of the toothbrushing diaries that generated little useable data.

Table 2: Self-report measures of toothbrushing pre- and post- intervention with effect estimate (95% CI)

HABIT

'Delivering Better Oral Health' (DBOH) guidelines	Baseline (n=27)	Two-week follow-up (n=24)*	Three-month follow-up (n=25)**
Using ≥1000ppm fluoride toothpaste	81% (0.66, 0.96)	100% (1.00, 1.00)	100% (1.00, 1.00)
Using a smear of toothpaste	63% (0.45, 0.81)	88% (0.75, 1.00)	88% (0.75, 1.00)
Parental brushing	81% (0.66, 0.96)	100% (1.00, 1.00)	100% (1.00, 1.00)
No rinsing after brushing	59% (0.40, 0.78)	92% (0.88, 1.00)	88% (0.75, 1.00)
Brushing at least twice a day, including at night	56% (0.37, 0.75)	88% (0.75, 1.00)	88% (0.75, 1.00)
**Total compliance to all DBOH guidelines**	**30% (0.13, 0.47)**	**71% (0.53, 0.89)**	**68% (0.50, 0.86)**

*2 self-reported brushing data sets not available
**1 self-reported brushing data set not available

Dietary Habits

For peer review only

Supplementary Item 2 illustrates the median consumption of notable food and drink categories over the course of the study, along with the frequency key used for coding. The consumption of biscuits decreased (BL: 4, 3MFU: 2) and sweets increased (BL: 0, 3MFU: 1), however, there were no significant changes in infant diet after the intervention. Use of free flow cups increased from 41% (n=11) at baseline to 72% (n=18) at the final follow-up visit.

Plaque Scores

The research team fully completed 67 (85%) plaque scores. The most common reasons for incomplete plaque scores were behavioural challenges or the infant being asleep. There was an incremental decrease in plaque scores between baseline (42%, n=25; 95%CI 0.23 to 0.61), two-week follow-up (20%, n=21; 95%CI 0.03 to 0.37) and the three-month follow-up (19%, n=21; 95%CI 0.02 to 0.36). The statistically significant decrease in plaque scores is represented in Figure 3.

Videos of Toothbrushing

From 79 data collection opportunities, video recording of toothbrushing was available for 75% (n=59) of home visits. Reasons for no video recording were as follows: five (n=5) of dyads were not brushing at baseline; five babies were asleep at the time of their home visit (n=5); three parents could not find their child's toothbrush at the baseline visit (n=3), one parent declined to consent for videotaping (n=1), two missed visits (see Figure 2) and two technical issues with the video camera (n=2). Toothbrushing duration increased from an average of 36 seconds at baseline (n=18, SD=23.9) to 47 seconds at the final follow up visit (n=19, SD=23.6).

Inter Examiner Reliability

Inter examiner reliability of scoring for dental examinations and plaque scores was assessed with Fleiss' kappa, where a value of 0.85 showed that scores were very highly significantly different from results that would have been obtained at random (p<0.001).

Discussion

Recruitment and Retention

Adequate recruitment and retention of participants are key outcome measures of any feasibility study[21]. Progression criteria should ideally be outlined in the protocol[20]; however, recommendations were first suggested in 2016 and this implementation has taken time[36]. Having achieved 28% recruitment and 74% retention of Group B, it is reasonable to recommend a conditional progression.

For peer review only

The intended recruitment and retention of Group A was achieved and feasible, however, the research team encountered some challenges. Initially, six Health Visitors were recruited to Group A but after three staff members went on long term sick leave, a further five were recruited to allow intervention delivery to continue without delay. Since the transfer of health visiting to local authorities, councils have seen a £700 million cut to public health spending [37]. In the three years leading up to HABIT commencement, Health Visitor numbers dropped by nearly a quarter, with a concurrent rise in stress-related sickness [38]. This study has identified, to recruit in a suitable timescale and alleviate pressure on an already stretched workforce, it will be necessary to increase intended recruitment numbers and continue to include wider members of the Health Visiting team.

The family groups in Group B varied in ethnicity, education, and income, and demographics were representative of the local area's high level of deprivation. Critically, this feasibility study recruited a diverse cohort within communities with high levels of oral disease [25]. Nearly half of participants lived in the 10% most deprived areas of England, according to the Index of Multiple Deprivation [25]. The average age of a parent in the study was 31, which is older than the average age of a first-time parent in England (28.9 years) [39], but is an expected finding given that most children (n=17) in the study were not first-born.

Approximately 28% of invited parents (Group B) were recruited to baseline, reaching the target sample size. Recruitment took ten months, four months longer than expected. Initially, the recruitment strategy included Health Visitors phoning up parents following the mail out of information, but Health Visitors struggled with this additional activity over and above their clinical workload. Following feedback, a member of the NHS Trust Research and Development team took over all recruitment responsibilities. Group B had a retention rate of 74% (n=26) at the final data-collection visit, a comparable level to similarly structured feasibility papers[40]. The biggest dropout occurred after consent (n=8). Combining the baseline data collection and consent visit would streamline future study design and minimise attrition[40]. Parents with young children lead busy and complex lives, which can lead to communication challenges and last-minute cancellations, consequently, generous timelines can provide the required flexibility for home-based data collection.

Feasibility of HABIT delivery and data collection

Intervention delivery and data collection in the home setting were feasible. The intervention targeted home-based oral health behaviours, therefore collecting data in this environment provides greater insight into household practices and parent-child interactions. Telephoning parents and organising visits within a tight time-period posed challenges; however, reasons for missed visits were generally due to unavoidable factors, such as child sickness. The data collection method may have

For peer review only

discouraged participation for some, but this did not hamper anticipated recruitment rates. Families received a £10 voucher for each home visit, which helped participant retention.

Intervention Outcomes

Self-reported toothbrushing behaviours

As with any feasibility study using a small sample size, any suggestion of impact needs to be considered with caution. Nonetheless, we can report encouraging signs of improvement in optimum oral health behaviours following the HABIT intervention. At the final data collection visit, there was a significant improvement in toothbrushing behaviours as shown by 'total' compliance with guidance. There were small increases in compliance to individual items of the guideline, however, these were non-significant within themselves. Importantly, by the end of the study, all parents had commenced infant toothbrushing with a fluoride toothpaste.

'Total' compliance to optimal toothbrushing behaviours was 30% at baseline. A key finding from this feasibility study is the low level of compliance when the five-point guideline criteria is used[7]. Previous studies have shown much higher levels of compliance [41-43]; however, these studies did not use the full guideline criteria nor home-based data collection methods. Paper toothbrushing diaries were rarely completed by parents, therefore self-reported toothbrushing frequency in the questionnaire was used in the total compliance measure. A recent incentivised study found that children, on average, only brushed their teeth five times a week, using data transmitted from a Bluetooth-enabled electric toothbrush [44], providing a clear justification for innovative data collection methods. Other contemporary data-collection methods, such as SMS messages sent each evening by parents also show initial signs of potential; 53% of their sample reported toothbrushing every evening [45]. These methods inconjunction with the videos of toothbrushing may provide a more accurate measure of optimal toothbrushing behaviours.

Dietary Habits

There was little reported change in dietary habits over the course of the feasibility study. This is a positive finding, given there is often an increase in the consumption of sugary drinks and snacks during infancy, due to the weaning process and the increase in consumption of all food groups[46 47]. The continued intake of water and milk, as opposed to a transition to sweetened drinks, is also encouraging. Moreover, the use of 'free-flow' cups increased and valve-cups decreased, another important and recommended practice for infant feeding [7]

Plaque Scores

For peer review only

Plaque scores showed an encouraging and incremental decrease between baseline visit and follow-up visits, however, undertaking plaque scores posed some challenges in this younger cohort. There were behavioural and cooperation challenges; four plaque scores were partially completed and not included in statistical analysis. A study using a similar cohort of patients found that sensitivity and specificity of plaque scores was poor, although this was undertaken without the use of a disclosing agent [48].

Videos of Toothbrushing

Filming of toothbrushing was acceptable to nearly all participants (n=26), however eight video opportunities were missed owing to logistical factors, such as the infant being asleep. Active parental toothbrushing was short in duration (36 secs), but showed a positive increase following the HABIT intervention (47 secs). A separate pilot study of toddlers demonstrated similar findings; despite toothbrushing sessions lasting, on average, over two minutes, active toothbrushing occurred for less than half this time [49]. This suggests that routine parental supervision of toothbrushing falls far short of professional expectations. Further analysis of these videos is beyond the scope of this paper and will be reported separately.

Limitations

Both Health Visitors and parent-child dyads were recruited through positive response to a research invitation. It could be assumed that they represent a more motivated and 'aware' subsection of the larger population, and thus a potential selection bias. To improve this in any larger trial or study, incentives, such as monetary gifts, pre-paid to participants, could be used to aid recruitment and retention in underserved groups [50]. Interpreting services were not available to the research team, which will have limited the inclusion of participants with restricted English.

Collecting information on infant dietary habits posed several difficulties. The questionnaire, although based on a substantiated data collection method [33] had shortcomings: it relied on memory, parents were sometimes unsure of diet when looked after by family and friends, and anecdotally there was confusion around the definition of some food groups. Amendment of this data collection method prior to a larger scale trial or study will be required. Certain dietary behaviours may be more easily and accurately collected through a focus on high-risk behaviours, such as sugar consumption in the hour before bedtime [51].

Conclusion

For peer review only - http://bmjopen.bmj.com/site/about/guidelines.xhtml

This early-phase feasibility study has demonstrated that HABIT is a feasible oral health intervention for Health Visitors to deliver to the parents of children aged 9-12-month-old. Data collection and intervention delivery in the home setting was achievable. Challenges relating to recruitment, collecting dietary data and retention of participants prior to baseline data collection need further modification prior to progression to a definite study or trial. The quantitative findings have shown an improvement in optimal oral health behaviours, suggesting an early signal of impact in a deprived population at high risk of caries.

For peer review only

Declarations

Ethics approval and consent to participate:

Ethical approval was obtained by the Research Ethics Committee (17/YH/0301) (Yorkshire & The Humber - Leeds East Research Ethics Committee), Health Research Authority (IRAS ID 230315) and NIHR CRN Portfolio Adoption. All methods were carried out in accordance with relevant guidelines and regulations.

Competing interests:

None declared.

Funding:

The study was funded by the Medical Research Council (MR/P017185/1). Four of the authors of this paper (PD, RM, ZM, and KGB) are supported by the NIHR Applied Research Collaborations Yorkshire and Humber (NIHR ARC YH) NIHR200166 www.arc-yh.nihr.ac.uk.

Author contributions:

PD is the principal investigator for the grant and led the development and writing of the trial protocol. EG led the analysis and writing of the manuscript with major contributions from RW, PD and KGB. EG, AB, FW, JO and IE undertook data collection, data cleaning and monitoring aspects of the protocol. All authors have read, contributed, and approved the final version of the manuscript. We would like to acknowledge our Health Visiting colleague, Victoria Smith, who co-authored the HABIT qualitative paper. It was our intention that Victoria would contribute in a similar manner to the HABIT quantitative analysis, however, she was unavailable to contribute to the final drafting of the paper.

Acknowledgements:

We would like to thank parents and health visiting team members who participated in the study as well as Susan Hanslip, Helen Gledhill, Jane Holt and Kerina Tull for their help with data collection. The views expressed in this publication are those of the author(s) and not necessarily those of the NIHR, the NHS or the Department of Health and Social Care. Further support for this feasibility study was provided by DenTCRU (Dental Translational and Clinical Research Unit), part of the NIHR Leeds Clinical Research Facility.

Data availability statement: The datasets generated and analysed during the current study are available upon request from Dr. Peter Day, p.f.day@leeds.ac.uk. These include transcripts of qualitative interviews and spreadsheets with the quantitative data. The data is kept for 20 years. Videos of child-parent toothbrushing are not available owing to the ethical assurances made to participants at the time of consent.

For peer review only

References

1. Global Burden of Disease Study. Global, regional, and national incidence, prevalence, and years lived with disability for 354 diseases and injuries for 195 countries and territories, 1990–2017: a systematic analysis for the Global Burden of Disease Study 2017. *Lancet* 2018;392:1789–8583. doi: https://doi.org/10.1016/S0140-6736(18)32279-7
2. Public Health England. Oral health survey of 5-year-old children 2019. https://www.gov.uk/government/statistics/oral-health-survey-of-5-year-old-children-2019, 2020.
3. Baghdadi ZD. Children's oral health-related quality of life and associated factors: mid-term changes after dental treatment under general anesthesia. *Journal of Clinical and Experimental Dentistry* 2015;7:327-38. doi: doi:10.4317/jced.51906
4. Gilchrist F, Marshman Z, Deery C, et al. The impact of dental caries on children and young people: what they have to say? *International journal of paediatric dentistry* 2015;25(5):327-38.
5. Gomes MC, de Almeida Pinto-Sarmento TC, de Brito Costa EMM, et al. Impact of oral health conditions on the quality of life of preschool children and their families: a cross-sectional study. *Health quality of life outcomes* 2014;12(1):55.
6. Broadbent JM, Thomson WM, Boyens JV, et al. Dental plaque and oral health during the first 32 years of life. *J Am Dent Assoc* 2011;142(4):415-26. [published Online First: 2011/04/02]
7. Public Health England. Delivering better oral health: an evidence-based toolkit for prevention 2017 [Available from: https://assets.publishing.service.gov.uk/government/uploads/system/uploads/attachment_data/file/605266/Delivering_better_oral_health.pdf.
8. Local Government Association. Health Visiting: giving children the best start in life. London: Local Government Association, 2019:1-30.
9. Department of Health and Social Care. Government response to the Health and Social Care Select Committee report on 'First 1000 days of life'. In: Department of Health and Social Care, ed. https://www.gov.uk/government/publications/government-response-to-the-first-1000-days-of-life-report/government-response-to-the-health-and-social-care-select-committee-report-on-first-1000-days-of-life: GOV UK, 2019.
10. Walker SP, Wachs TD, Gardner JM, et al. Child development: risk factors for adverse outcomes in developing countries. *Lancet* 2007;369(9556):145-57. doi: 10.1016/s0140-6736(07)60076-2 [published Online First: 2007/01/16]
11. HM Government. A Vision for the 1,001 Critical Days - The Best Start for Life. Early Years Health Development Review, 2021.
12. Public Health England. Rapid Review to Update Evidence for the Healthy Child Programme 0–5. London: https://www.gov.uk/government/uploads/system/uploads/attachment_data/file/429740/150520RapidReviewHealthyChildProg_UPDATE_poisons_final.pdf, 2021.
13. AlQahtani SJ, Hector MP, Liversidge HM. The London Atlas of Human Tooth Development and Eruption. *American journal of physical anthropology* 2010;142:481-90. doi: 10.1002/ajpa.21258
14. Coll AM, Filipponi T, Richards W. Health visitors' and school nurses' perceptions of promoting dental health in children. *Journal of Health Visiting* 2016;4(2):100-07.
15. Coll AM, Richards W, Filipponi T. Exploring attitudes of parents and teachers towards promoting oral health in children. *Journal of Health Visiting* 2017;5(8):402-09.
16. Oge OA, Douglas GV, Seymour D, et al. Knowledge, attitude and practice among Health Visitors in the United Kingdom toward children's oral health. *J Public Health Nursing* 2018;35(1):70-77.
17. Weston-Price S, Csikar J, Vinall-Collier K, et al. Barriers and facilitators to health visiting teams delivering oral health promotion to families of young children: a mixed methods study with vignettes. *Community Dental Health* 2020;37:1-9.

18. Eskyte I, Gray-Burrows K, Wray F, et al. Barriers to oral health conversations between Health Visitors and parents of children aged 9-12 months old. *Frontiers of Public Health* 2021;In preparation
19. Gray-Burrows K, Day P, Marshman Z, et al. Using intervention mapping to develop a home-based parental-supervised toothbrushing intervention for young children. *Implementation science* 2016;11(1):61.
20. Eskytė I, Gray-Burrows K, Owen J, et al. HABIT-an early phase study to explore an oral health intervention delivered by health visitors to parents with young children aged 9-12 months: study protocol. *Pilot Feasibility Stud* 2018;4:68. doi: 10.1186/s40814-018-0261-0 [published Online First: 2018/04/04]
21. MRC. Developing and evaluating complex interventions: new guidance. www.mrc.ac.uk/complexinterventionsguidance, 2006.
22. Craig P, Dieppe P, Macintyre S, et al. Developing and evaluating complex interventions: the new Medical Research Council guidance. *BMJ* 2008;337:a1655. doi: 10.1136/bmj.a1655
23. Bhatti A, Wray F, Eskytė I, et al. HABIT (Health Visitors delivering Advice in Britain on Infant Toothbrushing): A qualitative exploration of the acceptability of a complex oral health intervention". *BMC Family Practice (in submission)* 2021
24. Hoffmann TC, Glasziou PP, Boutron I, et al. Better reporting of interventions: template for intervention description and replication (TIDieR) checklist and guide. *BMJ* 2014;348:g1687. doi: 10.1136/bmj.g1687
25. Office of National Statistics. The English Indicies of Deprivation 2019. In: Ministry of Housing CaLG, ed. Online, 2019.
26. Office of National Statistics. Estimates of the population for the UK, England and Wales, Scotland and Northern Ireland. https://www.ons.gov.uk/peoplepopulationandcommunity, 2020.
27. Lancaster GA, Dodd S, Williamson PR. Design and analysis of pilot studies: recommendations for good practice. *Journal of Evaluation in Clinical Practice* 2004;10:307-12.
28. Pitts NB, Evans DJ, Pine CM. British Association for the Study of Community Dentistry (BASCD) diagnostic criteria for caries prevalence surveys-1996/97. *Community Dent Health* 1997;14 Suppl 1:6-9. [published Online First: 1997/03/01]
29. Greene JC, Vermilion JR. The Simplified Oral Hygiene Index. *J Am Dent Assoc* 1964;68:7-13. [published Online First: 1964/01/01]
30. Collett BR, Huebner CE, Seminario AL, et al. Observed child and parent toothbrushing behaviors and child oral health. *International journal of paediatric dentistry / the British Paedodontic Society [and] the International Association of Dentistry for Children* 2015 doi: 10.1111/ipd.12175
31. Mahoney S, Bryant M, Sahota P, et al. Dietary intake in the early years and its relationship to BMI in a bi-ethnic group: the Born in Bradford 1000 study. *Public Health Nutr* 2018;21(12):2242-54. doi: 10.1017/s1368980018000654 [published Online First: 2018/04/03]
32. Health and Social Care Information Centre. Child Dental Health Survey 2013, England, Wales and Northern Ireland [NS]. http://www.hscic.gov.uk/catalogue/PUB17137, 2015.
33. Dickerson J, Bird PK, McEachan RR, et al. Born in Bradford's Better Start: an experimental birth cohort study to evaluate the impact of early life interventions. *BMC Public Health* 2016;15:711. doi: 10.1186/s12889-016-3318-0
34. A language and environment for statistical computing. [program]. Vienna, Austria: R Foundation for Statistical Computing, 2020.
35. Bates D, Maechler M, Bolker B, et al. Fitting Linear Mixed-Effects Models Using lme4. *Journal of Statistical Software* 2015;67(1):1-48.
36. Eldridge SM, Chan CL, Campbell MJ, et al. CONSORT 2010 statement: extension to randomised pilot and feasibility trials. *Bmj* 2016;355:i5239. doi: 10.1136/bmj.i5239 [published Online First: 2016/10/26]

For peer review only - http://bmjopen.bmj.com/site/about/guidelines.xhtml

37. Institute of Health Visiting. Health Visiting in England: State of Health Visiting in England. https://ihv.org.uk/wp-content/uploads/2020/02/State-of-Health-Visiting-survey-FINAL-VERSION-18.2.20.pdf, 2020.
38. NHS Digital. Absence days due to stress, rate and proportion by region April 17 to March 2018. In: Digital N, ed., 2018.
39. NHS Digital. NHS Dental Statistics for England 2019-2020, biannual report. https://digital.nhs.uk/data-and-information/publications/statistical/nhs-dental-statistics/2019-20-biannual-report, 2020.
40. Giles E, Gray-Burrows KA, Bhatti A, et al. "Strong Teeth": an early-phase study to assess the feasibility of an oral health intervention delivered by dental teams to parents of young children. *BMC Oral Health* 2021;21(1):267. doi: 10.1186/s12903-021-01608-x [published Online First: 2021/05/19]
41. Wilson M, Morgan M. A survey of children's toothbrushing habits in Wales: Are parents following the guidelines? *Journal of Health Visiting* 2016;4(2):95-99. doi: 10.12968/johv.2016.4.2.95
42. Pine CM, Adair PM, Nicoll AD, et al. International comparisons of health inequalities in childhood dental caries. *Community dental health* 2004;21(1 Suppl):121-30.
43. Health Social Care Information Centre. Children's Dental Health Survey 2013: Health and Social Care Information Centre London, 2013.
44. White JS, Ramos-Gomez F, Liu JX, et al. Monetary incentives for improving smartphone-measured oral hygiene behaviors in young children: A randomized pilot trial. *PLoS One* 2020;15(7):e0236692. doi: 10.1371/journal.pone.0236692 [published Online First: 2020/07/31]
45. Kitsaras g. Bedtime routines: a dynamic, repetitive set of behaviours and their implications for child wellbeing, health and development. Manchester, 2019.
46. Schwartz C, Issanchou S, Nicklaus S. Developmental changes in the acceptance of the five basic tastes in the first year of life. *Br J Nutr* 2009;102(9):1375-85. doi: 10.1017/s0007114509990286 [published Online First: 2009/06/10]
47. Habibian M, Roberts G, Lawson M, et al. Dietary habits and dental health over the first 18 months of life. *Community Dent Oral Epidemiol* 2001;29(4):239-46. doi: 10.1034/j.1600-0528.2001.290401.x [published Online First: 2001/08/23]
48. Dumas SA, Weaver KE, Park SY, et al. Accuracy of visible plaque identification by pediatric clinicians during well-child care. *Clin Pediatr (Phila)* 2013;52(7):645-51. doi: 10.1177/0009922813483876 [published Online First: 2013/04/11]
49. Zeedyk MS, Longbottom C, Pitts NB. Tooth-brushing practices of parents and toddlers: a study of home-based videotaped sessions. *Caries Res* 2005;39(1):27-33. doi: 10.1159/000081653
50. Nicolaas G, Corteen E, Davis B. The use of incentives to recruit and retain hard-to-get populations in longitudinal studies: Economic and Social Research Council 2019.
51. Baghlaf K, Muirhead V, Moynihan P, et al. Free Sugars Consumption around Bedtime and Dental Caries in Children: A Systematic Review. *JDR Clin Trans Res* 2018;3(2):118-29. doi: 10.1177/2380084417749215 [published Online First: 2019/04/02]

For peer review only

Figure 1: Study Design

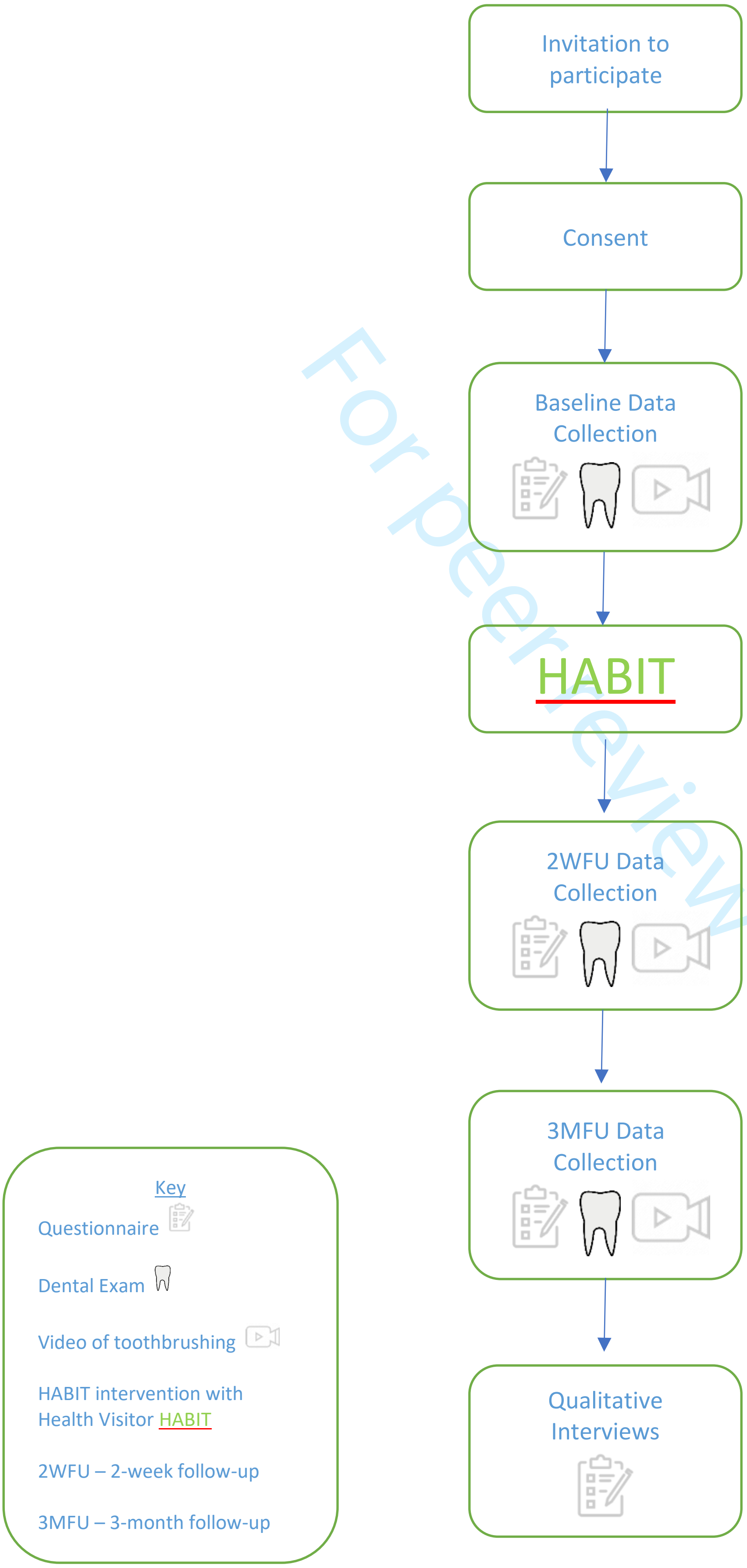

For peer review only

Figure 2: Participant flowchart, as outlined by CONSORT [35]

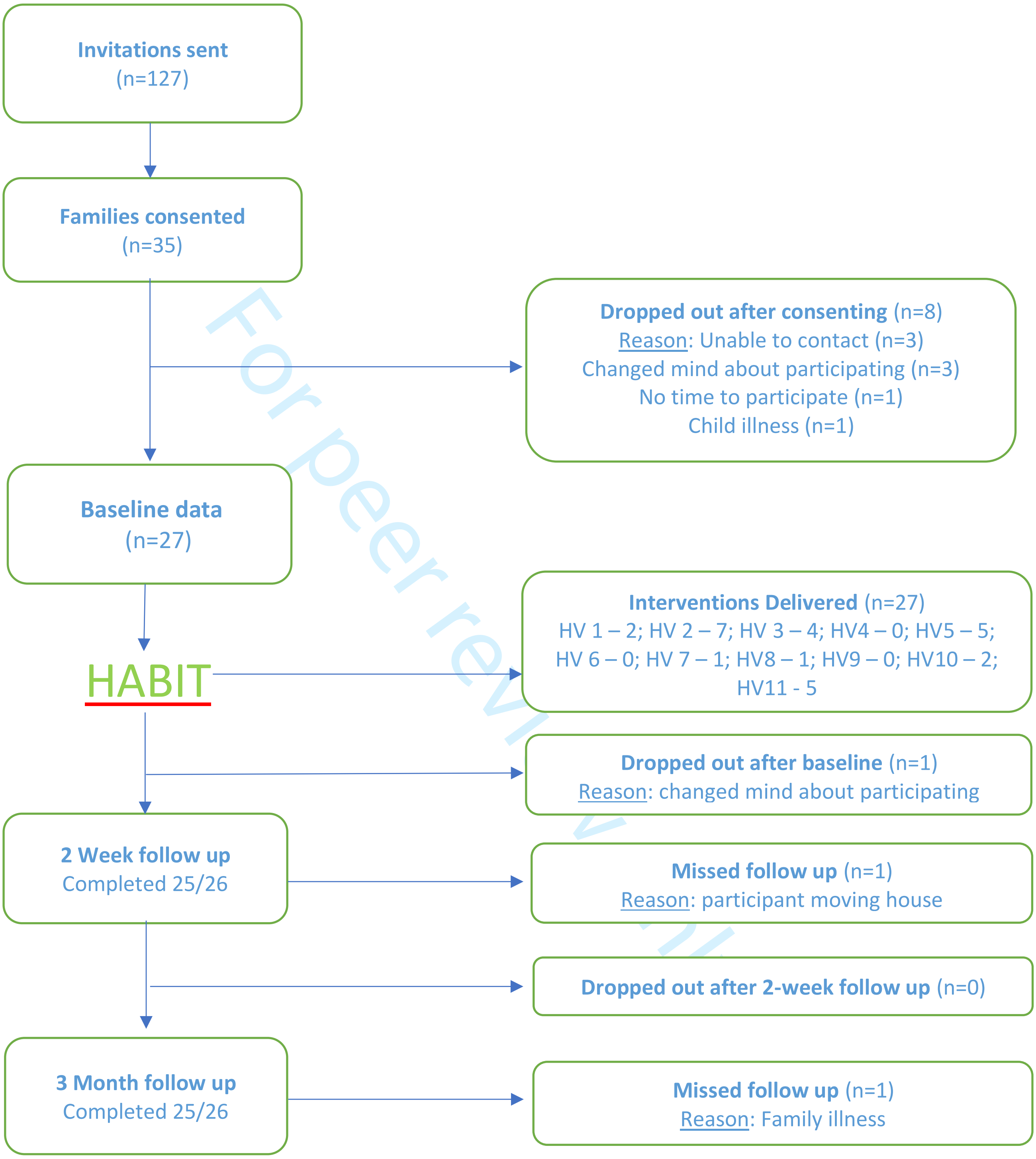

Figure 3: Plaque score trends between Baseline, 2WFU and 3MFU data collection visits

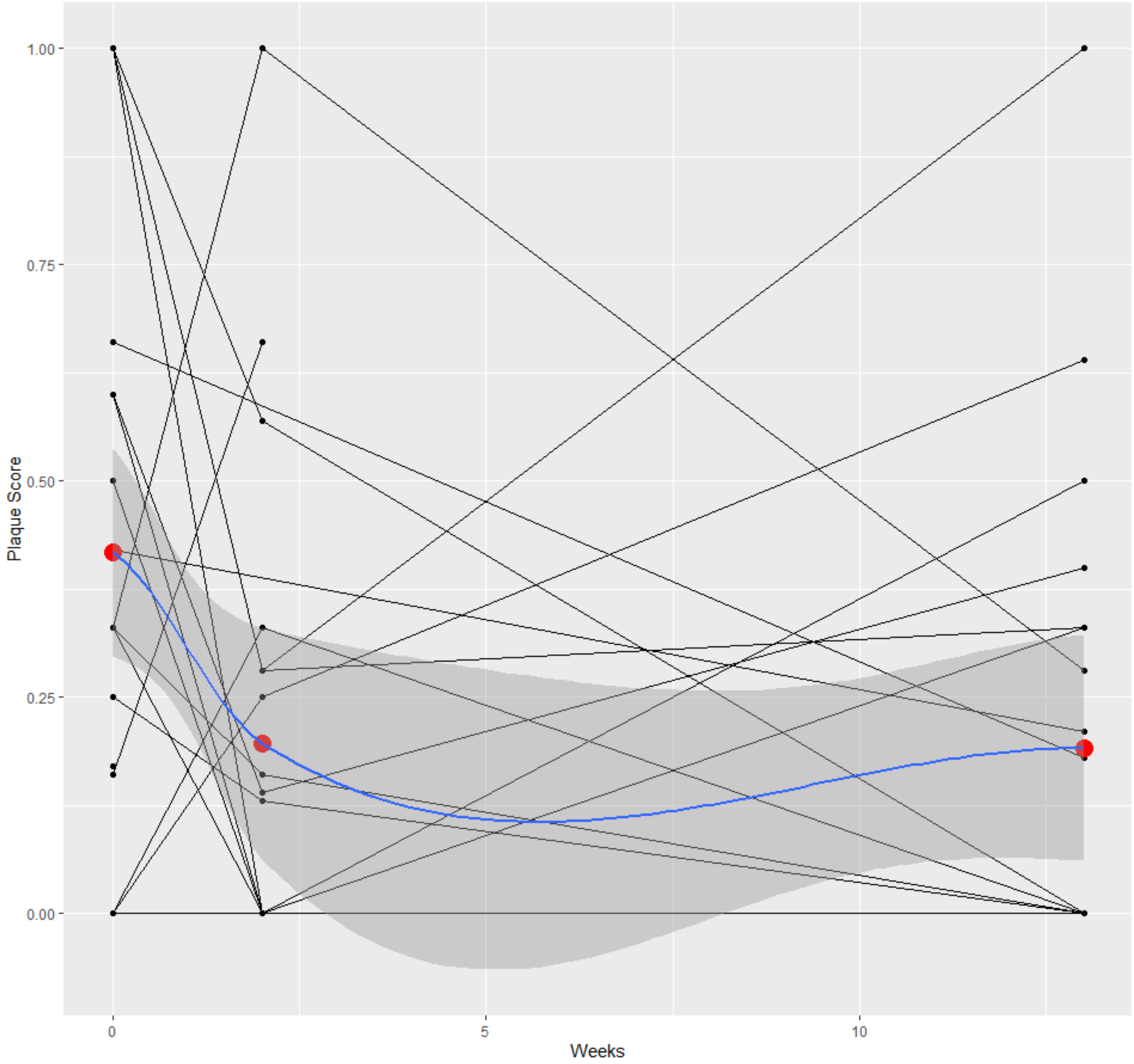

Supplementary Item 1: Intervention development using TIDieR (template for intervention description and replication) checklist (36).

Please note, the term, "health visitors" will be used collectively to represent health visitors and nursery nurses who took part in the HABIT intervention.

Brief name of intervention	HABIT (Health visitors delivering Advice in Britain on Infant Toothbrushing)
Why (rationale, theory, goal)	The impact of tooth decay is significant, with the disease affecting the child, their family and wider society. Health visitors and their teams are key public health professionals with an important role in providing advice and promoting health behaviours to families of young children. However, existing research has found wide variation in practice. Some health visitors have inconsistent access to training and limited practical knowledge on how to support parents overcome barriers to toothbrushing and limiting sugary foods and drinks.<br><br>HABIT is a co-designed complex intervention to support health visitors' oral health conversations with parents during the 9-12 month universal developmental home visit. The HABIT intervention delivered by health visitors, aims to empower parents to establish optimal oral health behaviours.<br><br>Following a complex intervention methodology, our multi-disciplinary team co-designed digital and paper-based training and resources with health visitors and parents of children aged 9–24 months. The intervention comprised of two components: (A) training for health visitors to deliver the HABIT intervention and (B) HABIT resources for parents, including a website, videos, toothbrushing demonstration and a paper-based leaflet with an action plan. Details on how the HABIT intervention was co-designed are described in Owen et al. (in preparation).
What<br>1. Materials for intervention and training (access to materials)<br>2. Procedures (describe activities and support activities)	1. All health visitors delivering the intervention attended a one-day training course. This included updates and guided discussions around oral health knowledge through viewing novel television-based programmes designed to support early-years professionals oral health knowledge (www.soap.media). The HABIT resource 'pack' was issued; including the standard delivery protocol (outlined below), HABIT leaflets, toothbrushing models, HABIT website link (www.toothbrushinghabit.com) and self-reported diaries. These different resources were reviewed and discussed with health visitors in conjunction with how to use them as part of an effective oral health conversation using tools from motivational interviewing. Behaviour change techniques were incorporated throughout the digital and paper-based HABIT resources.<br><br>2. At the first training event, health visitors, research team members and dental professionals agreed on a standard delivery protocol. This included: (1) Identifying parental concern about oral health and assessing the parent's motivation. (2) Engaging in an oral health conversation tailored to the parents needs and concerns using the HABIT leaflet as a guide. (3) Showing the HABIT website and appropriate video matched to parents' concern. (4) A toothbrushing demonstration (5) Developing a tailored action plan with the parent (5) Giving the parent the HABIT leaflet with a written action plan, toothbrush and toothpaste. (6) Completing health visitor diary.
Who provided (describe expertise, background, specific training)	The intervention is designed for delivery by health visiting teams (including health visitors and nursery nurses) following additional HABIT training.
How (modes of delivery, e.g., face to face/individual group)	HABIT is delivered as part of the Healthy Child Programme at the 9-12 month universal development review. The Healthy Child Programme is a universal intervention delivered by health visitors to all parents with young children aged 0-24 months. There are a minimum of five home visits of which, one is at the 9-12 month timepoint.
Where (types of locations)	Parental homes/clinics/children's centres
When and how much (how often is the intervention delivered, duration)	For purposes of the feasibility study, the HABIT intervention was delivered once.
Tailoring (how will the intervention be individualised)	The conversation is guided by parents, who self-identify barriers to oral health and solutions. Individualised action-plan is created.

For peer review only

Modifications (any changes during the study)	Reported throughout the manuscript.
How well<br>1. Intervention fidelity assessed by<br><br>2. Actual adherence	The results of the feasibility study are reported in two papers (Bhatti et al. 2021 and Giles et al. 2021). These papers described the acceptability of the HABIT intervention to parents and health visitors, the feasibility of delivery, and the intervention's impact on oral health behaviours of young children. A further paper (Owen et al. 2021) describes in detail the co-design of the HABIT intervention.<br><br>1. Fidelity was assessed through qualitative interviews and focus groups with parents and health visitors in conjunction with reviewing the diaries completed by health visitors following intervention delivery.<br><br>2. Actual adherence is reported in the qualitative paper reported in Bhatti et al. 2021.

For peer review only

Supplementary Item 2 – Dietary Data

Frequency of food and drinks averaged for all participants across the 3 data collection visits, coded using the following key: 0 - None; 1 - less than once a month; 2 - 1-3 time per month; 3 – once per week; 4 – 2-4 times per week; 5 – 5-6 times per week; 6 once per day; 7 – 2-3 times per day; 8 – 4-5 times per day; 9 – 6 or more times a day

	Median Frequency		
	Baseline (n=27)	2 Week Follow-Up (n=26)	3 Month Follow-Up (n=26)
Water	7	8	7
Milk	7	7	7
Sugared drinks	0	0	0
Cakes	0	0	0
Biscuits	4	4	2
Sweets	0	0	1
Fresh Fruit	7	7	7
Vegetables	7	7	6

For peer review only

HABIT: Health visitors delivering Advice in Britain on Infant Toothbrushing – An early-phase feasibility study of a complex oral health intervention

Journal:	*BMJ Open*
Manuscript ID	bmjopen-2021-059665.R1
Article Type:	Original research
Date Submitted by the Author:	27-Jul-2022
Complete List of Authors:	Giles, Erin; University of Leeds, School of Dentistry<br>Wray, Faye; University of Leeds, Leeds Institute of Health Sciences<br>Eskyte, Ieva; University of Leeds, School of Law<br>Gray-Burrows, Kara ; University of Leeds, School of Dentistry<br>Owen, Jenny; University of Leeds, School of Dentistry<br>Bhatti, Amrit; University of Leeds, School of Dentistry<br>Zoltie, Tim; University of Leeds, School of Dentistry<br>McEachan, Rosemary; Bradford Institute for Health Research<br>Marshman, Z; University of Sheffield Faculty of Medicine Dentistry and Health<br>Pavitt, Sue; University of Leeds, School of Dentistry<br>West, Robert; University of Leeds, Leeds Institute of Health Sciences<br>Day, Peter; University of Leeds, School of Dentistry
**Primary Subject Heading**:	Dentistry and oral medicine
Secondary Subject Heading:	Public health
Keywords:	PUBLIC HEALTH, Community child health < PAEDIATRICS, PREVENTIVE MEDICINE

I, the Submitting Author has the right to grant and does grant on behalf of all authors of the Work (as defined in the below author licence), an exclusive licence and/or a non-exclusive licence for contributions from authors who are: i) UK Crown employees; ii) where BMJ has agreed a CC-BY licence shall apply, and/or iii) in accordance with the terms applicable for US Federal Government officers or employees acting as part of their official duties; on a worldwide, perpetual, irrevocable, royalty-free basis to BMJ Publishing Group Ltd ("BMJ") its licensees and where the relevant Journal is co-owned by BMJ to the co-owners of the Journal, to publish the Work in this journal and any other BMJ products and to exploit all rights, as set out in our licence.

The Submitting Author accepts and understands that any supply made under these terms is made by BMJ to the Submitting Author unless you are acting as an employee on behalf of your employer or a postgraduate student of an affiliated institution which is paying any applicable article publishing charge ("APC") for Open Access articles. Where the Submitting Author wishes to make the Work available on an Open Access basis (and intends to pay the relevant APC), the terms of reuse of such Open Access shall be governed by a Creative Commons licence – details of these licences and which Creative Commons licence will apply to this Work are set out in our licence referred to above.

Other than as permitted in any relevant BMJ Author's Self Archiving Policies, I confirm this Work has not been accepted for publication elsewhere, is not being considered for publication elsewhere and does not duplicate material already published. I confirm all authors consent to publication of this Work and authorise the granting of this licence.

For peer review only

HABIT: Health visitors delivering Advice in Britain on Infant Toothbrushing – An early-phase feasibility study of a complex oral health intervention

Authors

Erin Giles, Faye Wray, Ieva Eskyte, Kara A Gray-Burrows, Jenny Owen, Amrit Bhatti, Tim Zoltie, Rosemary RC McEachan, Z Marshman, Sue Pavitt, Robert West, Peter Day

Corresponding Author

Erin Giles – Department of Paediatric Dentistry, School of Dentistry, University of Leeds, Leeds, LS2 9LU. Email: e.giles@leeds.ac.uk

Keywords

Dental caries, prevention, behaviour change conversations, oral health habits, dental team, health visitors, intervention, parents, feasibility, quantitative

Trial Registration

ISRCTN55332414

Word Count

4180

For peer review only

Abstract (Word Count: 293)

Objectives: to conduct an early-phase feasibility study of an oral health intervention, HABIT, delivered by Health Visitors to parents of children aged 9-12-months-old.

Design: a mixed-methods, early-phase, non-controlled, feasibility study.

Participants: recruitment consisted of Group A – HABIT-trained Health Visitors (n=11), and Group B - parents of children aged 9-12-months-old about to receive their universal health check (n=35).

Setting: Bradford, West Yorkshire, United Kingdom

Intervention: A multi-disciplinary team co-developed digital and paper-based training and resources with health visitors and parents of young children. The intervention comprised of two components: (A) training for health visitors to deliver the HABIT intervention and (B) HABIT resources for parents, including a website, videos, toothbrushing demonstration and a paper-based leaflet with an action plan.

Primary and Secondary Outcome Measures: Recruitment, retention, and intervention delivery were analysed as key process outcomes for Groups A & B. Group B demographics, self-reported toothbrushing behaviours, dietary habits and three objective measures of toothbrushing including plaque scores were collected at baseline, 2-weeks and 3-months post intervention.

Results: HABIT intervention delivery was feasible. Although the intended sample size was recruited (Group A=11; Group B=35) it was more challenging than anticipated. Retention of Group B participants to final data collection was satisfactory (n=26). Total compliance with toothbrushing guidelines at baseline was low (30%), but significantly improved and was maintained 3-months after the intervention (68%). Plaque scores improved post intervention and participants found video-recording of toothbrushing acceptable. Dietary habits remained largely unchanged.

Conclusion: This feasibility study has demonstrated that HABIT is an appropriate oral health intervention. Adaptions to the study design are recommended to maximise recruitment and data collection in a definitive study. These quantitative findings have demonstrated an early signal of impact for improved oral health behaviours for young children at high risk of decay.

Registration: ISRCTN55332414

For peer review only

Article Summary

Strengths and limitations of this study

- HABIT - a complex, co-designed oral health behavioural intervention – has been developed using MRC guidance.
- This early-phase feasibility study analysed recruitment, retention and intervention delivery as key process outcomes.
- Data were collected in the home setting and included clinical, observed and self-reported toothbrushing and dietary habit data collected at three time points: baseline and at two weeks and three months post HABIT intervention.
- Self-reporting of toothbrushing and dietary has limitations which the authors recognise.
- Challenges relating to recruitment and retention of participants will need to be addressed prior to progression to definitive study or full trial.

For peer review only

Background

Untreated dental caries is the most common health condition in the world. It is currently estimated that over half a billion children worldwide suffer from caries in their primary teeth (1). In England, a quarter of children have experienced caries by the age of five (2). However, wide disparities exist. Caries rates in deprived areas are around double the national average (2). The sequalae of untreated dental disease is significant with clear consequences to the child, their family and wider society – negative consequences include pain, infection, time off school, time off work for parents and a poorer quality of life (3-5).

Caries is a public health priority owing to its prevalence and impact. Critically, however, it is preventable. Optimal home-based oral health habits established in early childhood provide long-term protective benefits (6). National guidelines in England (7), provide clear, evidence-based definitions for these 'optimal' home-based oral health practices, including; brushing a child's teeth twice a day with a smear of at least 1000ppm fluoridated toothpaste as soon as the first tooth erupts into the mouth, and limiting the frequency and amount of sugary foods and drinks (7).

Health visiting teams in England provide a crucial role in giving children the best start possible in early-childhood by signposting families to support, encouraging positive health behaviours and safeguarding children at risk (8). Five mandatory universal visits are undertaken by the health visiting team during the first two years of each child's life, the fourth visit takes place when the child is aged 9-12-months-old (9). During this visit topics such as child development, health, nutrition, and obesity prevention are discussed. These visits can be undertaken by health visitors or appropriately trained nursery nurses. In this paper the term 'Health Visitors' will be used collectively to represent any health visiting team member who took part in the HABIT intervention.

The first 1000 days of a child's life are generally accepted to be the most important in their physical, psychological and emotional development (10, 11). As part of the Healthy Child Programme (12), oral health promotion is a key topic to be covered during these mandatory visits. The eruption of an infant's first tooth occurs at around six months of age (13), therefore the fourth universal health visit is usually the first opportunity for toothbrushing guidance to be discussed. Health Visitors, however, have identified barriers to provision of oral health advice including a lack of specialist knowledge, training, resources, navigating difficult conversations and recognising oral health as a priority (14-18).

"Health visitors delivering Advice in Britain on Infant Toothbrushing" (HABIT) is a co-designed complex oral health intervention, underpinned by behaviour change theory (19, 20). Feasibility studies are a critical step in the intervention development process; exploring acceptability, feasibility of delivery, recruitment and retention of participants and anticipating problems prior to a large-scale Randomised Control Trial (RCT) (21).

For peer review only - http://bmjopen.bmj.com/site/about/guidelines.xhtml

Aims and Objectives

The primary aim was to:

- Conduct an early-phase feasibility study of an oral health intervention, HABIT, delivered by Health Visitors to parents of children aged 9-12-months-old.

The objectives were to:

- Analyse recruitment and retention of participants of both Health Visitors and parents
- Assess the feasibility of intervention delivery and data collection
- Explore the potential impact of HABIT on optimal oral health behaviours

Method

Study design

This was a mixed-methods, early-phase, non-controlled, feasibility study. Health Visitors and Parents of children aged 9-12 months were recruited, all receiving the intervention.

Patient and Public Involvement

Participants were involved in the design, conduct, reporting and dissemination of this research project. Using a series of focus groups, previously designed health-visiting oral health resources were discussed and evaluated by Health Visitors and parents of young children. These discussions, in combination with the Medical Research Council (MRC) framework(22), informed the development of the intervention. A designated member of the research team supported and kept in regular contact with participants, allowing real-time feedback and alterations to the study conduct. Intervention and study design was acceptable to participants and is reported in a separate paper exploring the qualitative findings (23). In addition, key stakeholders were invited to a research dissemination event held in Bradford in which preliminary findings were presented and discussed. Participation included representatives from: Health Visiting and dental teams, Bradford District Care NHS Foundation Trust, Bradford Metropolitan District Council and the British Society of Paediatric Dentistry, Oral Health Strategy Group and Public Health England.

Intervention outline

HABIT is an oral health intervention, underpinned by complex behavioural change theory, undertaken in a home setting. It comprises of two parts: 1) Training Health Visitors to deliver the intervention; 2) Delivery of the HABIT intervention by trained Health Visitors at the 9–12-month universal health visit. Oral health conversations are supported by HABIT resources for parents, including a website, videos, toothbrushing demonstration and a paper-based leaflet with an action

For peer review only

plan. A detailed summary of the HABIT intervention is outlined using the TIDieR checklist (24) in Supplementary Item 1.

Setting

Bradford is a metropolitan borough located in the United Kingdom (UK), situated within the northern county of West Yorkshire. It is the fifth largest city in the UK with a population of approximately half a million. Bradford's urban areas are among the most deprived in the UK (25). The population of Bradford is also ethnically diverse – 64% of the population identify as White British, with 20% of the population of Pakistani ethnic-origin (26).

Recruitment & Retention

Group A participants were Health Visitors who provide the 9-12-month universal health check. Following grant funding, the research team worked with key contacts from the local health visiting team to disseminate research information. All eligible health visitors were invited to take part via email and those that volunteered to participate attended a one-day training course provided by the researchers.

Group B participants were recruited from the Bradford waiting list of children due to have their 9-12-month universal check. For each HABIT-trained Health Visitor, a report of their next 20 visits was identified. An invitation letter, project information sheet and consent form were posted with the standard universal check reminder letter. For Group B:

Inclusion criteria:

- Parents of a child aged 9-12-months-old who were about to receive a universal home visit by a HABIT-trained Health Visitor.
- Child with at least one erupted tooth

Exclusion criteria:

- Non-English-speaking families, as interpreter services were not available to the research team.

An NHS Trust Clinical Studies Officer contacted parents by phone to explore if they wanted to participate. If the invitation was accepted, they would visit the parent to gain written consent and organise a home visit for baseline data collection. As suggested in good practice recommendations (27), we intended to recruit 30 parents-children (dyads) to our feasibility study. Thirty participants would provide a 95% confidence interval (CI) for prevalence of no wider than ±19.6%, anticipating a minimum of 15% loss to follow-up.

For peer review only

Data collection

Data collection was carried out by three dental professionals experienced in providing care for children (SH, JO, HG), supported by three research assistants (KT, IE, FW). An experienced British Association of the Study of Community Dentistry (BASCD) examiner provided training to ensure a consistent approach to inspection procedures, BASCD caries assessment criteria and tooth codes(28). Dental researchers underwent calibration for caries and plaque detection, with agreement calculated using Fleiss' kappa.

Data were collected in a home setting. Children were examined supine, using a disposable dental mirror and a head torch for illumination. Cotton rolls were available, if required, to remove debris. Between the baseline and second data collection visit, parents received the HABIT intervention delivered by a Health Visitor as part of the child's universal 9-12-month health visit. Further data collection was carried out two-weeks and three-months following the intervention. A flowchart of study design is outlined in Figure 1.

1) First visit – baseline (BL). Self-reported toothbrushing and dietary behaviours were collected through a questionnaire. Toothbrushing diaries were issued as an additional, contemporaneous, measure of toothbrushing frequency. Three objective measures of parental supervised brushing (PSB) were collected:

    a) Teeth were examined for cavitated dentinal caries and restorations using the BASCD criteria (28).
    b) Children's pre-brushing plaque levels per sextant using the Oral Hygiene Index (29)
    c) Duration of toothbrushing: researchers filmed parent-child toothbrushing for subsequent evaluation (30)

2) Second visit – two-week follow-up (2WFU)

   Two weeks following the HABIT intervention, further self-report questionnaire data and objective measures of PSB (a-c above) were collected at a home visit.

3) Third visit – three-month follow-up (3MFU)

   Three months following the HABIT intervention, further self-report questionnaire data and objective measures of PSB (a-c above) were collected at a home visit.

A £10 gift voucher was issued after each home-based data collection visit.

Self-reported oral health behaviours

For peer review only

The validated questionnaire(31, 32) collected information on participant sociodemographic data, self-reported toothbrushing, dietary behaviours and feeding practices. Self-report determinants of toothbrushing were measured against national guidance(7) outlining five key items for toothbrushing: parental supervision, 1000ppm toothpaste fluoride concentration, smear of toothpaste, twice-daily toothbrushing and wiping away excess toothpaste at the end of brushing. A compound measure of 'total' compliance was also calculated, identifying those fully compliant to all five items. Contemporaneous data on the frequency of toothbrushing was collected by a paper diary. Dietary data were collected using an established questionnaire trialled within a Bradford birth cohort of similar aged children (33), measuring frequency of consumption of the main food groups. Frequency scoring was non-linear (e.g. 0=none, 1=less than once a month... 8=four-five times a day, 9=six times a day) and covered all aspects of diet with a focus on sugary snacks, sweetened beverages and fruit and vegetable intake.

Dental examination objective measures of toothbrushing

Teeth were examined for cavitated caries and dental restorations using the BASCD criteria (28). A score was calculated based on the number of decayed, missing and filled teeth (dmft). Prior to performing the Oral Hygiene Index, allergy status was confirmed (checking no allergy to food colourings) and petroleum jelly was applied to the lips. The infants' teeth were dyed using TePe PlaqSearch™ disclosing solution applied with a small dental brush. Once the plaque index (score) had been taken, gauze was used to wipe the dyed plaque from the teeth to ensure it did not act as a visual aid for the parent in the subsequent videotaping of toothbrushing. Partially completed plaque scores were discounted in the statistical analysis.

Videotaping of child

Videotaping of child-parent toothbrushing was undertaken using a small action camera (GoPro HERO 5, Go Pro Inc). Practicability of video recordings pertains to the feasibility of data collection and was therefore analysed alongside toothbrushing duration. The videos also provided an objective method of assessment with national toothbrushing guidelines (7) and will be reported in a separate paper.

For peer review only

Data Analysis

Data relating to recruitment, retention, intervention delivery and feasibility of data collection were analysed using descriptive statistics in MS Excel. Progression criteria to definitive study were agreed: >25% recruitment and >75% retention - automatic progression; 20-25% recruitment and 65-74% retention - modifications recommended; <20% recruitment and <64% retention - consultation with advisory group and remedial actions required.

To explore potential oral health impacts, confidence intervals (CIs) for continuous variables (e.g. plaque scores) were calculated using a t-distribution. For categorical outcomes, the CIs for rate were calculated based on the binomial distribution. In respect to self-reported toothbrushing compliance, repeated measurements were fitted to a multi-level model with timepoints nested within dyads and allocating a random intercept for dyad. Maximum likelihood fitting was used rather than reduced maximum likelihood to permit hypothesis testing. Within the model, a term was added for 'time points' increasing the degrees of freedom by two. This enabled the reporting of compliance rates at each time point and formal statistical testing of the effect of the intervention over time.

The statistical significance of the time term was then determined by use of the log-likelihood ratio test. Similarly, a two-level linear regression was fitted for plaque scores and changes over time tested through a log-likelihood test. This analysis was valid under the assumption that drop out was at random. The analysis was undertaken in the R statistical software environment (34) and using the lme4 package (35).

For peer review only

Results

Recruitment and Retention

Eleven Health Visitors were recruited to Group A (n=11) to deliver the HABIT intervention. Of the eleven, eight (73%) including six health visitors and two nursery nurses, had the opportunity to deliver the intervention, as outlined in Figure 2.

Group B dyads were recruited over a 10-month period between December 2017 and October 2018. From 127 invitations, 35 dyads were recruited and consented - a recruitment rate of 28%. Anticipating a minimum of 15% loss to follow-up, the achieved sample size of n=35 was sufficient. Nine participants were lost, resulting in a retention rate of 74% (n=26). Almost all dropouts occurred between consent (n=8) and prior to baseline data collection. Figure 2 shows the participant flow and reasons for dropout for Group B participants.

Demographics

The baseline demographic data is shown in Table 1. There was significant variation in education, employment status and family group types. Almost half (n=12) of the dyads lived in the most deprived decile area of England, according to the Index of Multiple Deprivation (IMD) 2019 (25). The average age of a parent in the study was 31, and the majority (n=21) were born in the United Kingdom. No child in the study had caries, an unsurprising finding given the age of the children.

Table 1: Baseline demographic data

Characteristic	N (%)
Age of Parent	
Mean	31 (6.0)
Range	19, 42
% over 30	19 (70)
Birthplace	
United Kingdom	21 (78)
	3(11)
Pakistan	
Other	3(11)
Number of children	
Multi-child house	17 (63)
Qualifications	
Less than 5 GCSEs	8 (30)
A-level or equivalent	10 (37)
University degree	9 (33)
Employment	
Currently employed	13 (48)
Household Income	
Less than £16100	3 (11)
£16100 to £ 21249	7 (26)
£21250 - £27999	1 (4)
£28000 - £38399	
£38399 +	2 (7)
No Answer/Unsure	5 (19)
	9 (33)
Finances	
'Living Comfortably'	11 (41)
'Doing Alright'	8 (29)
'Just Getting By'	1 (4)
'Finding It Difficult'	
	0 (0)
No Answer/Unsure	7 (26)
IMD Centile	
1st Most Deprived	12 (44)
2nd	2 (7)
3rd	4 (15)
4th	4 (15)
5th	2 (7)
6th	2 (7)
7th	1 (4)
8th	0 (0)
9th	0 (0)
10th Least Deprived	0 (0)

For peer review only

Feasibility of data collection and intervention delivery

Out of a possible 79 home visits, the research team were able to undertake 97% (n=77) of planned data collection. Reasons for the two missed visits are outlined in Figure 2. Of the 27 dyads who completed baseline data collection, all received the HABIT intervention.

Intervention Outcomes

Self-reported toothbrushing behaviours

For self-reported toothbrushing habits, 'total' compliance improved from 30% (n=27; 95%CI 0.13 to 0.47) at baseline to 70% (n=24; 95%CI 0.53 to 0.89) two-weeks and 68% (n=25; 95%CI 0.50 to 0.86) three-months following the HABIT intervention. These findings were statistically significant and are shown in Table 2. After the intervention, all parents had commenced toothbrushing. Compliance to all individual components of the guideline increased, albeit insignificantly, after the intervention and are outlined in Table 2. There was poor uptake of the toothbrushing diaries that generated little useable data.

Table 2: Self-report measures of toothbrushing pre- and post- intervention with effect estimate (95% CI)

'Delivering Better Oral Health' (DBOH) guidelines	Baseline N (%) ((95% C.I.))	Two-week follow-up* N (%) ((95% C.I.))	Three-month follow-up** N (%) ((95% C.I.))
Using ≥1000ppm fluoride toothpaste	22 (81) ((0.66, 0.96))	24 (100) ((1.00, 1.00))	25 (100) ((1.00, 1.00))
Using a smear of toothpaste	17 (63) (0.45, 0.81))	21 (88) ((0.75, 1.00))	22 (88) ((0.75, 1.00))
Parental brushing	22 (81) ((0.66, 0.96))	24 (100) ((1.00, 1.00))	25 (100) ((1.00, 1.00))
No rinsing after brushing	16 (59) ((0.40, 0.78))	22 (92) ((0.88, 1.00))	22 (88) ((0.75, 1.00))
Brushing at least twice a day, including at night	15 (56) ((0.37, 0.75))	22 (88) ((0.75, 1.00))	22 (88) ((0.75, 1.00))
**Total compliance to all DBOH guidelines**	**8 (30) ((0.13, 0.47))**	**17 (71) ((0.53, 0.89))**	**17 (68) ((0.50, 0.86))**

HABIT

*2 self-reported brushing data sets not available

**1 self-reported brushing data set not available

For peer review only

Dietary Habits

Supplementary Item 2 illustrates the median consumption of notable food and drink categories over the course of the study, along with the frequency key used for coding. The consumption of biscuits decreased (BL: 4, 3MFU: 2) and sweets increased (BL: 0, 3MFU: 1), however, there were no significant changes in infant diet after the intervention. Use of free flow cups increased from 41% (n=11) at baseline to 72% (n=18) at the final follow-up visit.

Plaque Scores

The research team fully completed 67 (85%) plaque scores. The most common reasons for incomplete plaque scores were behavioural challenges or the infant being asleep. There was an incremental decrease in plaque scores between baseline (42%, n=25; 95%CI 0.23 to 0.61), two-week follow-up (20%, n=21; 95%CI 0.03 to 0.37) and the three-month follow-up (19%, n=21; 95%CI 0.02 to 0.36). The statistically significant decrease in plaque scores is represented in Figure 3.

Videos of Toothbrushing

From 79 data collection opportunities, video recording of toothbrushing was available for 75% (n=59) of home visits. Reasons for no video recording were as follows: five (n=5) of dyads were not brushing at baseline; five babies were asleep at the time of their home visit (n=5); three parents could not find their child's toothbrush at the baseline visit (n=3), one parent declined to consent for videotaping (n=1), two missed visits (see Figure 2) and two technical issues with the video camera (n=2). Toothbrushing duration increased from an average of 36 seconds at baseline (n=18, SD=23.9) to 47 seconds at the final follow up visit (n=19, SD=23.6).

Inter Examiner Reliability

Inter examiner reliability of scoring for dental examinations and plaque scores was assessed with Fleiss' kappa, where a value of 0.85 showed that scores were very highly significantly different from results that would have been obtained at random (p<0.001).

Discussion

Recruitment and Retention

Adequate recruitment and retention of participants are key outcome measures of any feasibility study(21). Progression criteria should ideally be outlined in the protocol(20); however, recommendations were first suggested in 2016 and this implementation has taken time(36). Having achieved 28% recruitment and 74% retention of Group B, it is reasonable to recommend a conditional progression.

For peer review only

The intended recruitment and retention of Group A was achieved and feasible, however, the research team encountered some challenges. Initially, six Health Visitors were recruited to Group A but after three staff members went on long term sick leave, a further five were recruited to allow intervention delivery to continue without delay. Since the transfer of health visiting to local authorities, councils have seen a £700 million cut to public health spending (37). In the three years leading up to HABIT commencement, Health Visitor numbers dropped by nearly a quarter, with a concurrent rise in stress-related sickness (38). This study has identified, to recruit in a suitable timescale and alleviate pressure on an already stretched workforce, it will be necessary to increase intended recruitment numbers and continue to include wider members of the Health Visiting team.

Caries predominantly affects lower-socioeconomic areas of England, therefore Bradford was an ideal location for the implementation of oral health research. The family groups in Group B varied in ethnicity, education, and income, and demographics were representative of the local area's high level of deprivation. Critically, this feasibility study recruited a diverse cohort within communities with high levels of oral disease (25). Nearly half of participants lived in the 10% most deprived areas of England, according to the Index of Multiple Deprivation (25). The average age of a parent in the study was 31, which is older than the average age of a first-time parent in England (28.9 years) (39), but is an expected finding given that most children (n=17) in the study were not first-born.

Approximately 28% of invited parents (Group B) were recruited to baseline, reaching the target sample size. Recruitment took ten months, four months longer than expected. Initially, the recruitment strategy included Health Visitors phoning up parents following the mail out of information, but Health Visitors struggled with this additional activity over and above their clinical workload. Following feedback, a member of the NHS Trust Research and Development team took over all recruitment responsibilities. Group B had a retention rate of 74% (n=26) at the final data-collection visit, a comparable level to similarly structured feasibility papers(40). The biggest dropout occurred after consent (n=8). Combining the baseline data collection and consent visit would streamline future study design and minimise attrition(40). Parents with young children lead busy and complex lives, which can lead to communication challenges and last-minute cancellations, consequently, generous timelines can provide the required flexibility for home-based data collection.

Feasibility of HABIT delivery and data collection

Intervention delivery and data collection in the home setting were feasible. The intervention targeted home-based oral health behaviours, therefore collecting data in this environment provides greater insight into household practices and parent-child interactions. Telephoning parents and organising visits within a tight time-period posed challenges; however, reasons for missed visits were generally due to unavoidable factors, such as child sickness. The data collection method may have

For peer review only

discouraged participation for some, but this did not hamper anticipated recruitment rates. Families received a £10 voucher for each home visit, which helped participant retention.

Intervention Outcomes

Self-reported toothbrushing behaviours

As with any feasibility study using a small sample size, any suggestion of impact needs to be considered with caution. Nonetheless, we can report encouraging signs of improvement in optimum oral health behaviours following the HABIT intervention. At the final data collection visit, there was a significant improvement in toothbrushing behaviours as shown by 'total' compliance with guidance. There were small increases in compliance to individual items of the guideline, however, these were non-significant within themselves. Importantly, by the end of the study, all parents had commenced infant toothbrushing with a fluoride toothpaste.

'Total' compliance to optimal toothbrushing behaviours was 30% at baseline. A key finding from this feasibility study is the low level of compliance when the five-point guideline criteria is used(7). Previous studies have shown much higher levels of compliance (41-43); however, these studies did not use the full guideline criteria nor home-based data collection methods. Paper toothbrushing diaries were rarely completed by parents, therefore self-reported toothbrushing frequency in the questionnaire was used in the total compliance measure. A recent incentivised study found that children, on average, only brushed their teeth five times a week, using data transmitted from a Bluetooth-enabled electric toothbrush (44), providing a clear justification for innovative data collection methods. Other contemporary data-collection methods, such as SMS messages sent each evening by parents also show initial signs of potential; 53% of their sample reported toothbrushing every evening (45). These methods in conjunction with the videos of toothbrushing may provide a more accurate measure of optimal toothbrushing behaviours.

To date, there is a lack of published studies in the UK looking at the effectiveness of oral health interventions in young children (46, 47) . A recent Australian study has found that both telephone and home contacts by an oral therapist were effective at reducing caries experience and cost-effective when compared to routine care, although caution should be exerted in relation to the generalisability to a UK population(48). A previous RCT found no significant difference in caries experience between trained health visitor oral heal, however, methodological limitations and interference between study groups restrict the applicability of the findings(49).

Dietary Habits

There was little reported change in dietary habits over the course of the feasibility study. This is a positive finding, given there is often an increase in the consumption of sugary drinks and snacks during infancy, due to the weaning process and the increase in consumption of all food groups(50,

For peer review only

51). The continued intake of water and milk, as opposed to a transition to sweetened drinks, is also encouraging. Moreover, the use of 'free-flow' cups increased and valve-cups decreased, another important and recommended practice for infant feeding (7)

Plaque Scores

Plaque scores showed an encouraging and incremental decrease between baseline visit and follow-up visits, however, undertaking plaque scores posed some challenges in this younger cohort. There were behavioural and cooperation challenges; four plaque scores were partially completed and not included in statistical analysis. A study using a similar cohort of patients found that sensitivity and specificity of plaque scores was poor, although this was undertaken without the use of a disclosing agent (52).

Videos of Toothbrushing

Filming of toothbrushing was acceptable to nearly all participants (n=26), however eight video opportunities were missed owing to logistical factors, such as the infant being asleep. Active parental toothbrushing was short in duration (36 secs), but showed a positive increase following the HABIT intervention (47 secs). A separate pilot study of toddlers demonstrated similar findings; despite toothbrushing sessions lasting, on average, over two minutes, active toothbrushing occurred for less than half this time (53). This suggests that routine parental supervision of toothbrushing falls far short of professional expectations.

Limitations

Both Health Visitors and parent-child dyads were recruited through positive response to a research invitation. It could be assumed that they represent a more motivated and 'aware' subsection of the larger population, and thus a potential selection bias. To improve this in any larger trial or study, incentives, such as monetary gifts, pre-paid to participants, could be used to aid recruitment and retention in underserved groups (54). Interpreting services were not available to the research team, which will have limited the inclusion of participants with restricted English. Liaising and working with parents closely will be key to the success of any future study. Arranging visits at a time when the infant is most likely to cooperate, such as avoiding nap time, will maximise the utility of plaques scores and videotaping.

Collecting information on infant dietary habits posed several difficulties. The questionnaire, although based on a substantiated data collection method (33) had shortcomings: it relied on memory, parents were sometimes unsure of diet when looked after by family and friends, and anecdotally

For peer review only

there was confusion around the definition of some food groups and dietary terminology (e.g. no 'added' sugar compared to sugar-free). Amendment of this data collection method prior to a definitive trial. Certain dietary behaviours cam be more easily and accurately collected through a focus on high-risk behaviours, such as sugar consumption in the hour before bedtime (55).

Conclusion

This early-phase feasibility study has demonstrated that HABIT is a feasible oral health intervention for Health Visitors to deliver to the parents of children aged 9-12-month-old. Data collection and intervention delivery in the home setting was achievable. Challenges relating to recruitment, collecting dietary data and retention of participants prior to baseline data collection need further modification prior to progression to a definite study or trial. The quantitative findings have shown an improvement in optimal oral health behaviours, suggesting an early signal of impact in a deprived population at high risk of caries.

For peer review only

Declarations

Ethics approval and consent to participate:

Ethical approval was obtained by the Research Ethics Committee (17/YH/0301) (Yorkshire & The Humber - Leeds East Research Ethics Committee), Health Research Authority (IRAS ID 230315) and NIHR CRN Portfolio Adoption. All methods were carried out in accordance with relevant guidelines and regulations.

Competing interests:

None declared.

Funding:

The study was funded by the Medical Research Council (MR/P017185/1). Four of the authors of this paper (PD, RM, ZM, and KGB) are supported by the NIHR Applied Research Collaborations Yorkshire and Humber (NIHR ARC YH) NIHR200166 www.arc-yh.nihr.ac.uk.

Author contributions:

PD is the principal investigator for the grant and led the development and writing of the trial protocol. EG led the analysis and writing of the manuscript with major contributions from RW, PD and KGB. EG, AB, FW, JO and IE undertook data collection, data cleaning and monitoring aspects of the protocol. All authors including RM, ZM, TZ and SP have had the opportunity to read, contribute, and approve the manuscript.

Acknowledgements:

We would like to thank parents and health visiting team members including Susanne Gill, who participated in the study as well as Susan Hanslip, Helen Gledhill, Jane Holt and Kerina Tull for their help with data collection. We would like to acknowledge our Health Visiting colleagues, Victoria Smith, who co-authored the HABIT qualitative paper. It was our intention that Victoria would contribute in a similar manner to the HABIT quantitative analysis, however, she was unavailable to contribute to the final drafting of the paper. The views expressed in this publication are those of the author(s) and not necessarily those of the NIHR, the NHS or the Department of Health and Social Care. Further support for this feasibility study was provided by DenTCRU (Dental Translational and Clinical Research Unit), part of the NIHR Leeds Clinical Research Facility.

Data availability statement: The datasets generated and analysed during the current study are available upon request from Dr. Peter Day, p.f.day@leeds.ac.uk. These include transcripts of qualitative interviews and spreadsheets with the quantitative data. The data is kept for 20 years. Videos of child-parent toothbrushing are not available owing to the ethical assurances made to participants at the time of consent.

References

1. Global Burden of Disease Study. Global, regional, and national incidence, prevalence, and years lived with disability for 354 diseases and injuries for 195 countries and territories, 1990–2017: a systematic analysis for the Global Burden of Disease Study 2017. Lancet. 2018;392:1789–8583.

2. Public Health England. Oral health survey of 5-year-old children 2019. https://www.gov.uk/government/statistics/oral-health-survey-of-5-year-old-children-2019; 2020.
3. Baghdadi ZD. Children's oral health-related quality of life and associated factors: mid-term changes after dental treatment under general anesthesia. Journal of Clinical and Experimental Dentistry. 2015;7:327-38.
4. Gilchrist F, Marshman Z, Deery C, Rodd HD. The impact of dental caries on children and young people: what they have to say? International journal of paediatric dentistry. 2015;25(5):327-38.
5. Gomes MC, de Almeida Pinto-Sarmento TC, de Brito Costa EMM, Martins CC, Granville-Garcia AF, Paiva SMJH, et al. Impact of oral health conditions on the quality of life of preschool children and their families: a cross-sectional study. Health quality of life outcomes. 2014;12(1):55.
6. Broadbent JM, Thomson WM, Boyens JV, Poulton R. Dental plaque and oral health during the first 32 years of life. J Am Dent Assoc. 2011;142(4):415-26.
7. Public Health England. Delivering better oral health: an evidence-based toolkit for prevention 2017 [Available from: https://assets.publishing.service.gov.uk/government/uploads/system/uploads/attachment_data/file/605266/Delivering_better_oral_health.pdf.
8. Local Government Association. Health Visiting: giving children the best start in life. London: Local Government Association; 2019. p. 1-30.
9. Department of Health and Social Care. Government response to the Health and Social Care Select Committee report on 'First 1000 days of life'. In: Department of Health and Social Care, editor. https://www.gov.uk/government/publications/government-response-to-the-first-1000-days-of-life-report/government-response-to-the-health-and-social-care-select-committee-report-on-first-1000-days-of-life: GOV UK; 2019.
10. Walker SP, Wachs TD, Gardner JM, Lozoff B, Wasserman GA, Pollitt E, et al. Child development: risk factors for adverse outcomes in developing countries. Lancet. 2007;369(9556):145-57.
11. HM Government. A Vision for the 1,001 Critical Days - The Best Start for Life. 2021.
12. Public Health England. Rapid Review to Update Evidence for the Healthy Child Programme 0–5. London: https://www.gov.uk/government/uploads/system/uploads/attachment_data/file/429740/150520RapidReviewHealthyChildProg_UPDATE_poisons_final.pdf; 2021.
13. AlQahtani SJ, Hector MP, Liversidge HM. The London Atlas of Human Tooth Development and Eruption. American journal of physical anthropology. 2010;142:481-90.
14. Coll AM, Filipponi T, Richards W. Health visitors' and school nurses' perceptions of promoting dental health in children. Journal of Health Visiting. 2016;4(2):100-7.
15. Coll AM, Richards W, Filipponi T. Exploring attitudes of parents and teachers towards promoting oral health in children. Journal of Health Visiting. 2017;5(8):402-9.
16. Oge OA, Douglas GV, Seymour D, Adams C, Csikar J. Knowledge, attitude and practice among Health Visitors in the United Kingdom toward children's oral health. J Public Health Nursing. 2018;35(1):70-7.
17. Weston-Price S, Csikar J, Vinall-Collier K, Bishop P, D'Antoni D, Pine C. Barriers and facilitators to health visiting teams delivering oral health promotion to families of young children: a mixed methods study with vignettes. Community Dental Health. 2020;37:1-9.
18. Eskyte I, Gray-Burrows K, Wray F, Owen J, Pavitt S, West R, et al. Barriers to oral health conversations between Health Visitors and parents of children aged 9-12 months old. Frontiers of Public Health. 2021;In preparation.
19. Gray-Burrows K, Day P, Marshman Z, Aliakbari E, Prady S, McEachan R. Using intervention mapping to develop a home-based parental-supervised toothbrushing intervention for young children. Implementation science. 2016;11(1):61.

For peer review only - http://bmjopen.bmj.com/site/about/guidelines.xhtml

20. Eskytė I, Gray-Burrows K, Owen J, Sykes-Muskett B, Zoltie T, Gill S, et al. HABIT-an early phase study to explore an oral health intervention delivered by health visitors to parents with young children aged 9-12 months: study protocol. Pilot Feasibility Stud. 2018;4:68.
21. MRC. Developing and evaluating complex interventions: new guidance. www.mrc.ac.uk/complexinterventionsguidance; 2006.
22. Craig P, Dieppe P, Macintyre S, Michie S, Nazareth I, Petticrew M, et al. Developing and evaluating complex interventions: the new Medical Research Council guidance. BMJ. 2008;337:a1655.
23. Bhatti A, Wray F, Eskytė I, Gray-Burrows KA, Owen J, Giles E, et al. HABIT (Health Visitors delivering Advice in Britain on Infant Toothbrushing): A qualitative exploration of the acceptability of a complex oral health intervention". BMC Family Practice (in submission). 2021.
24. Hoffmann TC, Glasziou PP, Boutron I, Milne R, Perera R, Moher D, et al. Better reporting of interventions: template for intervention description and replication (TIDieR) checklist and guide. BMJ. 2014;348:g1687.
25. Office of National Statistics. The English Indicies of Deprivation 2019. In: Ministry of Housing CaLG, editor. Online2019.
26. Office of National Statistics. Estimates of the population for the UK, England and Wales, Scotland and Northern Ireland. https://www.ons.gov.uk/peoplepopulationandcommunity; 2020.
27. Lancaster GA, Dodd S, Williamson PR. Design and analysis of pilot studies: recommendations for good practice. Journal of Evaluation in Clinical Practice. 2004;10:307-12.
28. Pitts NB, Evans DJ, Pine CM. British Association for the Study of Community Dentistry (BASCD) diagnostic criteria for caries prevalence surveys-1996/97. Community Dent Health. 1997;14 Suppl 1:6-9.
29. Greene JC, Vermilion JR. The Simplified Oral Hygiene Index. J Am Dent Assoc. 1964;68:7-13.
30. Collett BR, Huebner CE, Seminario AL, Wallace E, Gray KE, Speltz ML. Observed child and parent toothbrushing behaviors and child oral health. International journal of paediatric dentistry / the British Paedodontic Society [and] the International Association of Dentistry for Children. 2015.
31. Mahoney S, Bryant M, Sahota P, Barber S. Dietary intake in the early years and its relationship to BMI in a bi-ethnic group: the Born in Bradford 1000 study. Public Health Nutr. 2018;21(12):2242-54.
32. Health and Social Care Information Centre. Child Dental Health Survey 2013, England, Wales and Northern Ireland [NS]. http://www.hscic.gov.uk/catalogue/PUB17137; 2015.
33. Dickerson J, Bird PK, McEachan RR, Pickett KE, Waiblinger D, Uphoff E, et al. Born in Bradford's Better Start: an experimental birth cohort study to evaluate the impact of early life interventions. BMC Public Health. 2016;15:711.
34. R Core Team. A language and environment for statistical computing. . Vienna, Austria: R Foundation for Statistical Computing; 2020.
35. Bates D, Maechler M, Bolker B, Walker S. Fitting Linear Mixed-Effects Models Using lme4. Journal of Statistical Software. 2015;67(1):1-48.
36. Eldridge SM, Chan CL, Campbell MJ, Bond CM, Hopewell S, Thabane L, et al. CONSORT 2010 statement: extension to randomised pilot and feasibility trials. Bmj. 2016;355:i5239.
37. Institute of Health Visiting. Health Visiting in England: State of Health Visiting in England. https://ihv.org.uk/wp-content/uploads/2020/02/State-of-Health-Visiting-survey-FINAL-VERSION-18.2.20.pdf; 2020.
38. NHS Digital. Absence days due to stress, rate and proportion by region April 17 to March 2018. In: Digital N, editor. 2018.
39. NHS Digital. NHS Dental Statistics for England 2019-2020, biannual report. https://digital.nhs.uk/data-and-information/publications/statistical/nhs-dental-statistics/2019-20-biannual-report2020.

For peer review only

40. Giles E, Gray-Burrows KA, Bhatti A, Rutter L, Purdy J, Zoltie T, et al. "Strong Teeth": an early-phase study to assess the feasibility of an oral health intervention delivered by dental teams to parents of young children. BMC Oral Health. 2021;21(1):267.
41. Wilson M, Morgan M. A survey of children's toothbrushing habits in Wales: Are parents following the guidelines? Journal of Health Visiting. 2016;4(2):95-9.
42. Pine CM, Adair PM, Nicoll AD, Burnside G, Petersen PE, Beighton D, et al. International comparisons of health inequalities in childhood dental caries. Community dental health. 2004;21(1 Suppl):121-30.
43. Health Social Care Information Centre. Children's Dental Health Survey 2013. Health and Social Care Information Centre London; 2013.
44. White JS, Ramos-Gomez F, Liu JX, Jue B, Finlayson TL, Garza JR, et al. Monetary incentives for improving smartphone-measured oral hygiene behaviors in young children: A randomized pilot trial. PLoS One. 2020;15(7):e0236692.
45. Kitsaras g. Bedtime routines: a dynamic, repetitive set of behaviours and their implications for child wellbeing, health and development. Manchester: Manchester; 2019.
46. York Health Economics Consortium. A rapid review of evidence on the cost-effectiveness of interventions to improve the oral health of children aged 0-5 years 2016 [Available from: https://assets.publishing.service.gov.uk/government/uploads/system/uploads/attachment_data/file/560972/Rapid_review_ROI_oral_health_5_year_old.pdf.
47. Downer MC, Drugan CS, Blinkhorn AS. A critique of the Brushing for Life programme. Health Education Journal. 2006;65(1):84-92.
48. Koh R, Pukallus M, Kularatna S, Gordon LG, Barnett AG, Walsh LJ, et al. Relative cost-effectiveness of home visits and telephone contacts in preventing early childhood caries. Community Dentistry and Oral Epidemiology. 2015;43(6):560-8.
49. Whittle J, Whitehead H, Bishop C. A randomised control trial of oral health education provided by a health visitor to parents of pre-school children. Community dental health. 2008;25(1):28-32.
50. Schwartz C, Issanchou S, Nicklaus S. Developmental changes in the acceptance of the five basic tastes in the first year of life. Br J Nutr. 2009;102(9):1375-85.
51. Habibian M, Roberts G, Lawson M, Stevenson R, Harris S. Dietary habits and dental health over the first 18 months of life. Community Dent Oral Epidemiol. 2001;29(4):239-46.
52. Dumas SA, Weaver KE, Park SY, Polk DE, Weyant RJ, Bogen DL. Accuracy of visible plaque identification by pediatric clinicians during well-child care. Clin Pediatr (Phila). 2013;52(7):645-51.
53. Zeedyk MS, Longbottom C, Pitts NB. Tooth-brushing practices of parents and toddlers: a study of home-based videotaped sessions. Caries Res. 2005;39(1):27-33.
54. Nicolaas G, Corteen E, Davis B. The use of incentives to recruit and retain hard-to-get populations in longitudinal studies. Economic and Social Research Council 2019.
55. Baghlaf K, Muirhead V, Moynihan P, Weston-Price S, Pine C. Free Sugars Consumption around Bedtime and Dental Caries in Children: A Systematic Review. JDR Clin Trans Res. 2018;3(2):118-29.

Figure 1: Study Design

Figure 2: Participant flowchart, as outlined by CONSORT [35]

For peer review only

Figure 3: Plaque score trends between Baseline, 2WFU and 3MFU data collection visits

Supplementary Item 1: Intervention development using TIDieR (template for intervention description and replication) checklist (36).

Please note, the term, "health visitors" will be used collectively to represent health visitors and nursery nurses who took part in the HABIT intervention.

Supplementary Item 2: Dietary Data

Frequency of food and drinks averaged for all participants across the 3 data collection visits, coded using the following key:

0 - None; 1 - less than once a month; 2 - 1-3 time per month; 3 – once per week; 4 – 2-4 times per week; 5 – 5-6 times per week; 6 once per day; 7 – 2-3 times per day; 8 – 4-5 times per day; 9 – 6 or more times a day

For peer review only

Figure 1: Study Design

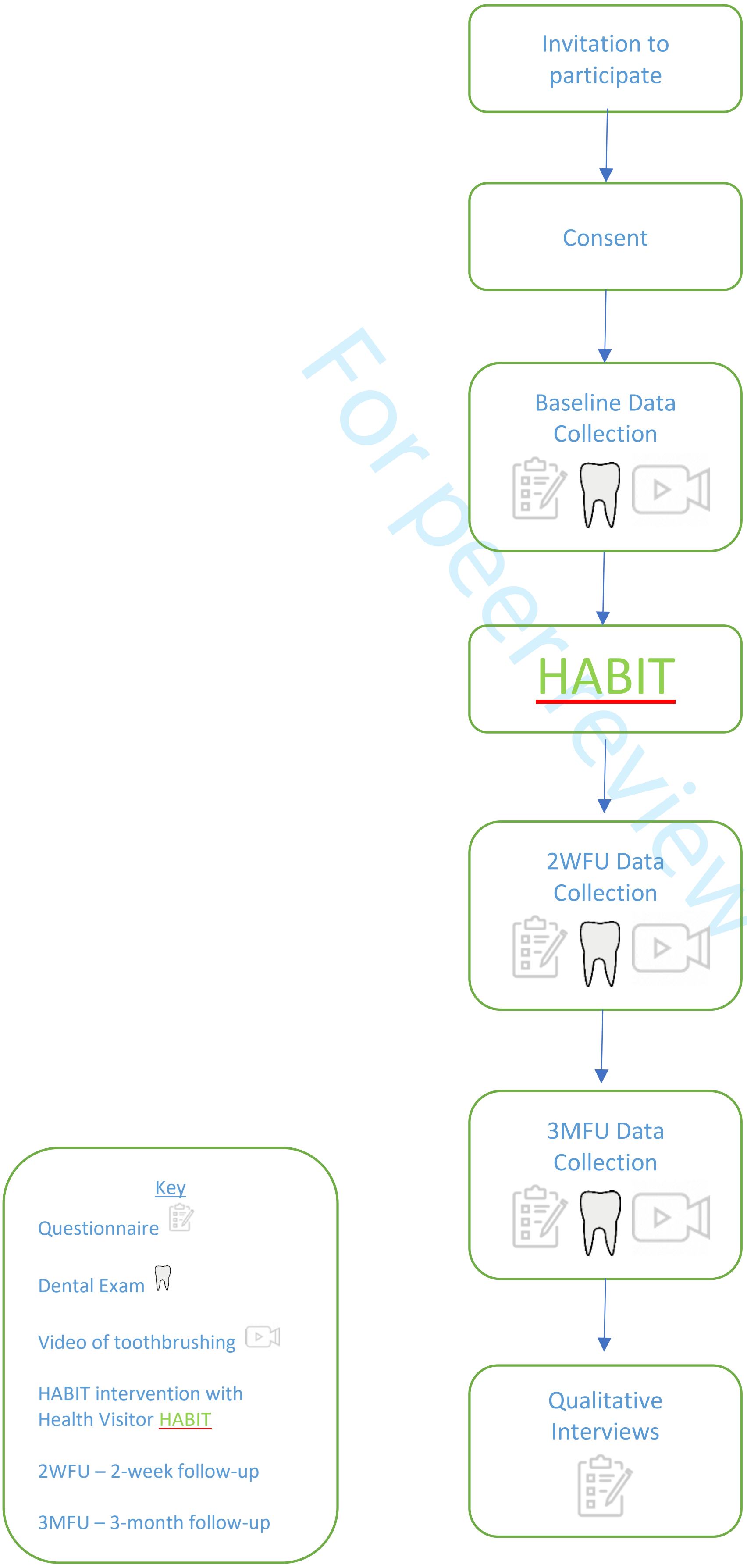

For peer review only

Figure 2: Participant flowchart, as outlined by CONSORT [35]

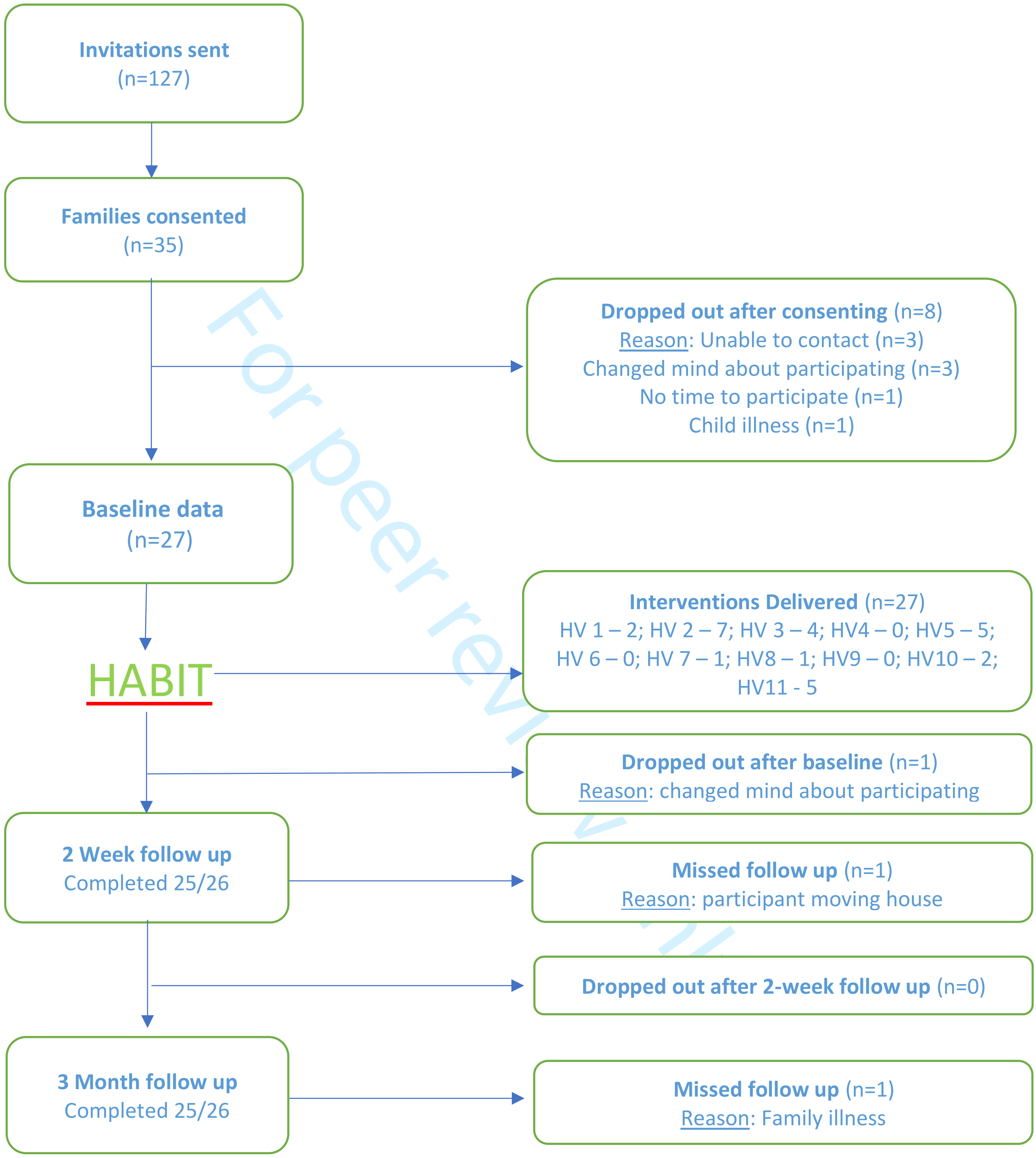

Figure 3: Plaque score trends between Baseline, 2WFU and 3MFU data collection visits

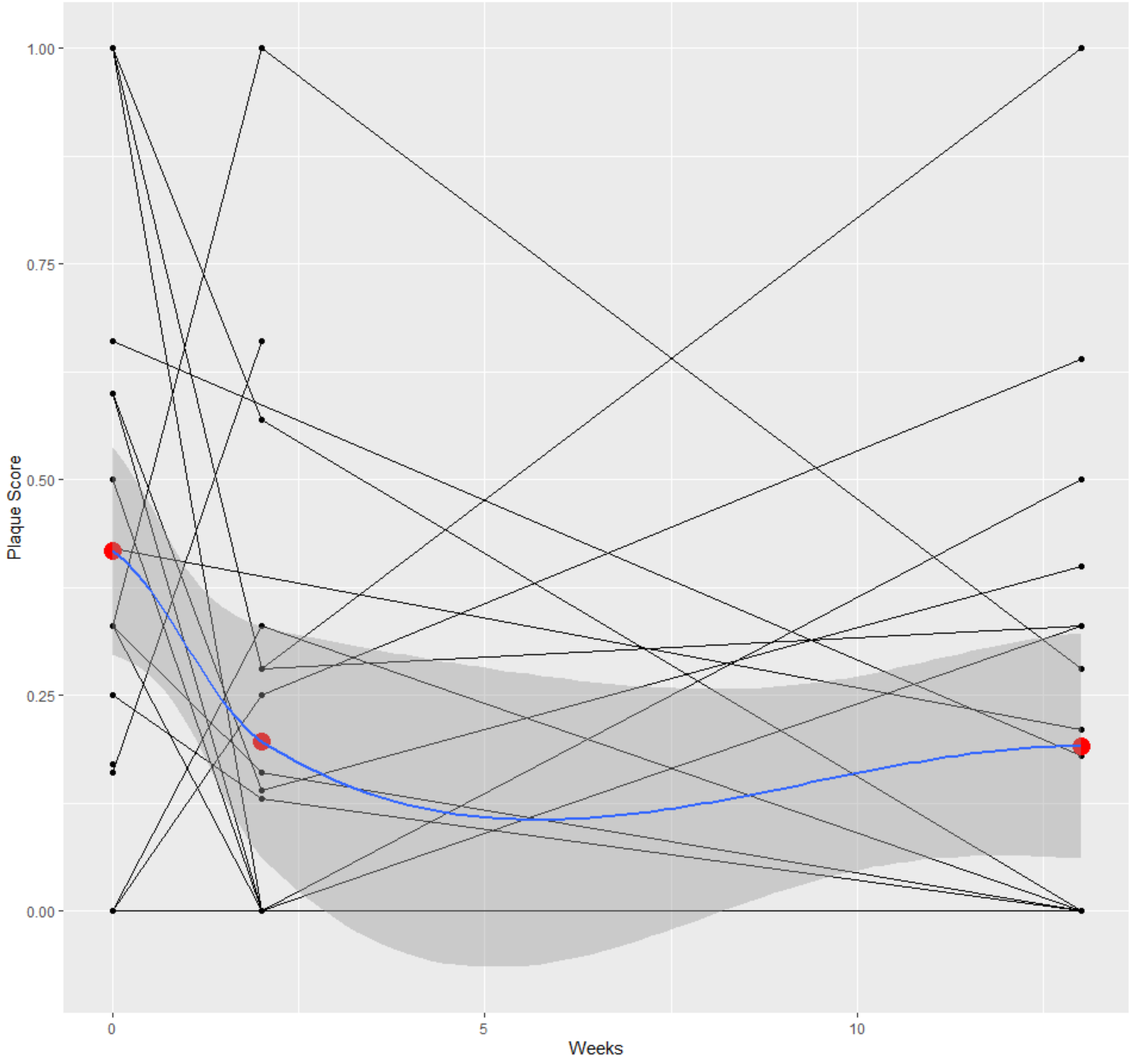

Supplementary Item 1: Intervention development using TIDieR (template for intervention description and replication) checklist (36).

Please note, the term, “health visitors” will be used collectively to represent health visitors and nursery nurses who took part in the HABIT intervention.

Brief name of intervention	HABIT (Health visitors delivering Advice in Britain on Infant Toothbrushing)
Why (rationale, theory, goal)	The impact of tooth decay is significant, with the disease affecting the child, their family and wider society. Health visitors and their teams are key public health professionals with an important role in providing advice and promoting health behaviours to families of young children. However, existing research has found wide variation in practice. Some health visitors have inconsistent access to training and limited practical knowledge on how to support parents overcome barriers to toothbrushing and limiting sugary foods and drinks.<br><br>HABIT is a co-designed complex intervention to support health visitors’ oral health conversations with parents during the 9-12 month universal developmental home visit. The HABIT intervention delivered by health visitors, aims to empower parents to establish optimal oral health behaviours.<br><br>Following a complex intervention methodology, our multi-disciplinary team co-designed digital and paper-based training and resources with health visitors and parents of children aged 9–24 months. The intervention comprised of two components: (A) training for health visitors to deliver the HABIT intervention and (B) HABIT resources for parents, including a website, videos, toothbrushing demonstration and a paper-based leaflet with an action plan. Details on how the HABIT intervention was co-designed are described in Owen et al. (in preparation).
What<br>1. Materials for intervention and training (access to materials)<br>2. Procedures (describe activities and support activities)	1. All health visitors delivering the intervention attended a one-day training course. This included updates and guided discussions around oral health knowledge through viewing novel television-based programmes designed to support early-years professionals oral health knowledge (www.soap.media). The HABIT resource ‘pack’ was issued; including the standard delivery protocol (outlined below), HABIT leaflets, toothbrushing models, HABIT website link (www.toothbrushinghabit.com) and self-reported diaries. These different resources were reviewed and discussed with health visitors in conjunction with how to use them as part of an effective oral health conversation using tools from motivational interviewing. Behaviour change techniques were incorporated throughout the digital and paper-based HABIT resources.<br><br>2. At the first training event, health visitors, research team members and dental professionals agreed on a standard delivery protocol. This included: (1) Identifying parental concern about oral health and assessing the parent’s motivation. (2) Engaging in an oral health conversation tailored to the parents needs and concerns using the HABIT leaflet as a guide. (3) Showing the HABIT website and appropriate video matched to parents’ concern. (4) A toothbrushing demonstration (5) Developing a tailored action plan with the parent (5) Giving the parent the HABIT leaflet with a written action plan, toothbrush and toothpaste. (6) Completing health visitor diary.
Who provided (describe expertise, background, specific training)	The intervention is designed for delivery by health visiting teams (including health visitors and nursery nurses) following additional HABIT training.
How (modes of delivery, e.g., face to face/individual group)	HABIT is delivered as part of the Healthy Child Programme at the 9-12 month universal development review. The Healthy Child Programme is a universal intervention delivered by health visitors to all parents with young children aged 0-24 months. There are a minimum of five home visits of which, one is at the 9-12 month timepoint.
Where (types of locations)	Parental homes/clinics/children’s centres
When and how much (how often is the intervention delivered, duration)	For purposes of the feasibility study, the HABIT intervention was delivered once.
Tailoring (how will the intervention be individualised)	The conversation is guided by parents, who self-identify barriers to oral health and solutions. Individualised action-plan is created.

For peer review only

Modifications (any changes during the study)	Reported throughout the manuscript.
How well<br>1. Intervention fidelity assessed by<br><br>2. Actual adherence	The results of the feasibility study are reported in two papers (Bhatti et al. 2021 and Giles et al. 2021). These papers described the acceptability of the HABIT intervention to parents and health visitors, the feasibility of delivery, and the intervention's impact on oral health behaviours of young children. A further paper (Owen et al. 2021) describes in detail the co-design of the HABIT intervention.<br><br>1. Fidelity was assessed through qualitative interviews and focus groups with parents and health visitors in conjunction with reviewing the diaries completed by health visitors following intervention delivery.<br><br>2. Actual adherence is reported in the qualitative paper reported in Bhatti et al. 2021.

For peer review only

Supplementary Item 2 – Dietary Data

Frequency of food and drinks averaged for all participants across the 3 data collection visits, coded using the following key: 0 - None; 1 - less than once a month; 2 - 1-3 time per month; 3 – once per week; 4 – 2-4 times per week; 5 – 5-6 times per week; 6 once per day; 7 – 2-3 times per day; 8 – 4-5 times per day; 9 – 6 or more times a day

	Median Frequency		
	Baseline (n=27)	2 Week Follow-Up (n=26)	3 Month Follow-Up (n=26)
Water	7	8	7
Milk	7	7	7
Sugared drinks	0	0	0
Cakes	0	0	0
Biscuits	4	4	2
Sweets	0	0	1
Fresh Fruit	7	7	7
Vegetables	7	7	6

For peer review only
